# Enhancing vanadium pentoxide-based (V_2_O_5_) cathodes for high-performance aqueous zinc-ion batteries: optimization of interlayer spacing, ion kinetics, voltage window

**DOI:** 10.1039/d5ra04247j

**Published:** 2025-09-19

**Authors:** Patient D. Nzengu, Ntuthuko W. Hlongwa, Kutloano E. Sekhosana, Mesfin A. Kebede

**Affiliations:** a Institute for Nanotechnology and Water Sustainability (iNanoWS), Engineering and Technology, University of South Africa Roodepoort, Florida, 1710 Johannesburg Gauteng South Africa mesfiak@unisa.ac.za

## Abstract

The urgent need for safe, affordable, and environmentally responsible energy storage has placed rechargeable aqueous zinc-ion batteries (AZIBs) at the centre of next-generation research. While earlier reviews surveyed V_2_O_5_ cathodes in broad terms, none has yet unified the latest insights on interlayer engineering, electrolyte coordination, and *operando* diagnostics into a coherent design framework. Focusing on progress from 2019 to mid-2025, this review offers three distinctive contributions. First, it correlates the crystallographic evolution of V_2_O_5_, captured by synchrotron X-ray, *in situ* TEM, and Raman studies, with voltage plateaus and capacity decay, providing a mechanistic map of Zn^2+^/H_2_O co-intercalation and phase transitions. Second, it compares emerging synthesis routes (sol–gel, hydrothermal, solid-state, and electrochemical deposition) through a quantitative lens, linking specific surface area, defect chemistry, and conductivity (10^−2^ to 10^−1^ S cm^−1^) to rate capability and long-term retention. Third, it surveys interlayer-expansion strategies, metal-ion pre-intercalation, organic pillar insertion, conductive-polymer hybrids, and hierarchical nanostructuring, showing how each modulates Zn^2+^ diffusivity, lattice strain (<5% with water co-insertion), and dissolution resistance. By integrating experimental advances with density-functional-theory, *ab initio* molecular-dynamics, and machine-learning predictions, the review distils actionable design principles and a forward roadmap for achieving >400 mAh g^−1^ capacities and >90% retention beyond 2000 cycles. These new perspectives position V_2_O_5_ not merely as a promising cathode, but as a model system for understanding and optimizing layered hosts in aqueous multivalent batteries.

## Abstract 2

The urgent need for safe, affordable, and environmentally responsible energy storage has placed rechargeable aqueous zinc-ion batteries (AZIBs) at the centre of next-generation research.

## Graphical abstract

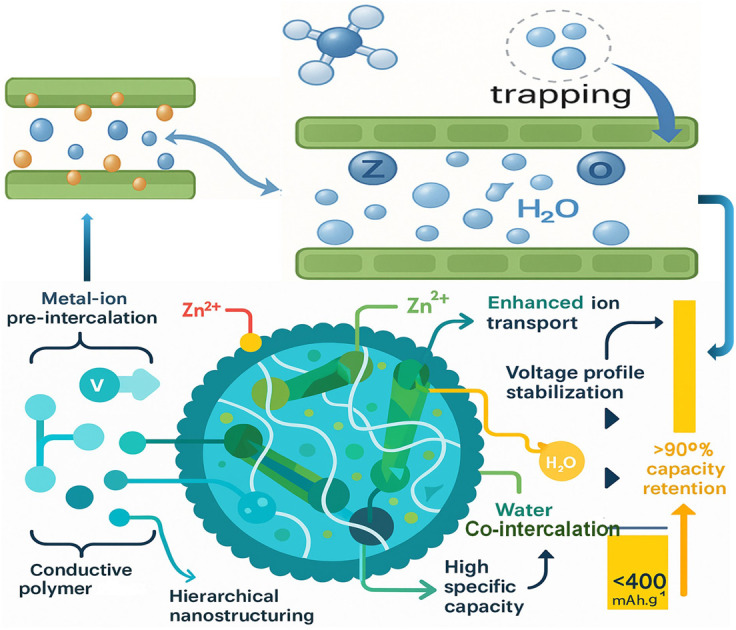

## 1. Introduction

The global demand for high-performance energy storage systems has catalysed extensive research into alternative chemistries beyond conventional lithium-ion batteries (LIBs), particularly those utilizing safer and more abundant materials. Among the emerging technologies, rechargeable aqueous zinc-ion batteries (AZIBs) have attracted substantial attention due to their inherent advantages such as high safety derived from non-flammable aqueous electrolytes, environmental sustainability, low manufacturing cost, and the high theoretical capacity of metallic zinc (820 mAh g^−1^, 5851 mAh cm^−3^) as an anode material.^1–3^ A critical barrier to the commercial viability of AZIBs, however, lies in the development of robust cathode materials capable of reversibly hosting Zn^2+^ ions in aqueous media without suffering from structural degradation or electrochemical.^3^ Vanadium pentoxide (V_2_O_5_), with its layered orthorhombic structure, high theoretical capacity (294–440 mAh g^−1^), and multivalent redox capability (V^5+^ ↔ V^4+^ ↔ V^3+^), stands out as a leading cathode candidate.^4,5^ Its open interlayer channels facilitate hydrated Zn^2+^ ions intercalation, while the polymorphism and tunable oxidation states of vanadium provide a versatile platform for electrochemical performance enhancement.^6–8^ Despite these merits, the performance of V_2_O_5_-based cathodes in aqueous media is hindered by several intrinsic limitations. The insertion of divalent Zn^2+^ ions, especially in hydrated form (radius ∼4.3 Å), exerts significant lattice strain often exceeding 5–10%, which accelerates structural collapse, phase transitions, and active material dissolution during cycling.^9–11^ Compounding this issue is the low intrinsic electronic conductivity of pristine V_2_O_5_ (∼10^−2^ S cm^−1^), which limits charge transport and rate capacity.^10^ These challenges necessitate deliberate structural and chemical modifications to enhance Zn^2+^ diffusion, suppress lattice degradation, and improve-term cycling stability. While prior reviews have broadly surveyed V_2_O_5_ cathodes and general strategies for performance enhancement, they often lack critical discussion on the role if interlayer engineering, voltage window optimization, and co-intercalation mechanisms, each of which is central to understanding the dynamic electrochemistry of V_2_O_5_ in aqueous systems. Furthermore, few have contextualized their discussions with quantitative metrics such as conductivity improvement, lattice strain mitigation, or interlayer spacing modulation.^12–14^

This review aims to address these gaps by offering a focused and critical evaluation of recent advances in the design and optimization of V_2_O_5_-based cathodes for AZIBs. Emphasis is placed on interlayer engineering strategies, including the pre-intercalation of metal ions, incorporation of water (H_2_O) molecules, and integration of polymeric matrices, that modulate interlayer spacing and enhance ion transport kinetics. We also analyse phase evolution pathways, voltage profiles, and capacity retention behaviours in details, linking these phenomena to structural and electronic features. By consolidating both mechanistic insights and quantitative performance data, this review provides a foundation for the rational design of next-generation V_2_O_5_ cathodes and outlines strategic directions for overcoming current performance bottlenecks in AZIB systems.

## 2. Fundaments of AZIBs

Commercial Zn-MnO_2_ battery systems often use alkaline electrolytes, which serve as the ion-conducting medium between the Zn anode and the MnO_2_ cathode and benefit from the relatively low redox potential of Zn(OH)_4_^2−^/Zn couple. However, these alkaline Zn-MnO_2_ cells are generally primary (non-rechargeable) batteries.^15^ In contrast, modern rechargeable AZIBs typically employ mildly acidic or near-neutral aqueous electrolytes to enable reversible Zn^2+^ deposition and dissolution. This distinction means that alkaline Zn-MnO_2_ systems should not be conflated with rechargeable AZIBs.

Aqueous zinc-ion battery technology features a zinc metal anode, an electrolyte (some containing zinc), and a cathode that serves as the host material for zinc ions during battery operation. This technology is considered promising for several reasons:

(1) *Electrolyte range*: it can utilize both aqueous and non-aqueous electrolytes, offering flexibility in design and application.

(2) *High redox potential*: zinc ions operate effectively in aqueous electrolytes due to their high redox potential (−0.763 V *vs.* SHE), a performance level not achievable with other battery systems such as SIBs, PIBs, and LIBs.

(3) *Enhanced safety and low toxicity*: zinc-ion batteries offer improved safety and reduced toxicity compared to some other battery technologies.

(4) *Reversibility of Zn plating/stripping*: zinc plating and stripping are reversible, particularly in neutral or slightly acidic electrolytes. In contrast, alkaline electrolytes can cause issues such as dendrite growth, corrosion due to by-products like ZnO, and hydrogen evolution reactions (HERs), which can be detrimental to the anode.^16^(Zn + 4OH^−^ → Zn(OH)_4_^2−^ + 2e^−^ → ZnO + 2OH^−^ + H_2_O + 2e^−^)

The AZIB system is a promising battery technology with the potential for greater cycling stability compared to other battery types. This is due to the high density of the zinc anode metal and the involvement of both electrons in the electrochemical reaction mechanisms. Additionally, the compact size of AZIBs makes them suitable for use in devices such as epidermal, implantable, and wearable sensors. Moreover, AZIBs can be produced on a large scale and commercialized effectively due to their advantageous characteristics and features.^17^

The design and scalability of AZIB technology largely depend on the performance of the chosen cathode materials. The open-circuit voltage, zinc ion storage capacity, and the underlying mechanisms are influenced by the properties of the cathode material. A well-designed cathode significantly affects the electrochemical performance of the battery system, including rate performance and gravimetric power density. This is because the cathode materials are crucial for ionic and electronic transport, as well as redox reactions occurring at the cathode and the cathode–electrolyte interfaces. Therefore, it is essential to develop a cathode with stable and integral structures, as the stability and integrity of the cathode directly impact the cycling stability of the battery system.^18^

### 2.1. Challenges associated with rechargeable aqueous zinc-ion batteries (AZIBs)

Despite the numerous advantages of AZIB technology, there are several challenges related to the electrodes, particularly the anode. This section focuses on these challenges:

Three primary interrelated issues are observed with the anode: dendrite growth, corrosion, and HERs.

*Dendrite growth*: this phenomenon occurs during charge–discharge cycles due to the uneven electric field distribution and a limited number of active sites on the anode. The resulting dendrite growth increases the internal resistance of the battery and may lead to short circuits or gas evolution posing serious safety risks and potentially damaging the separator.

*Corrosion*: corrosion is caused by the presence of active water molecules and leads to the liberation of hydrogen, which increases the internal pressure of the battery. This pressure can cause deformation of the battery's shape and possible electrolyte leakage, render the battery toxic and decrease its capacity by deactivating active sites on the zinc anode.

*Hydrogen evolution reactions (HERs)*: HERs contribute to the internal pressure and subsequent corrosion, further affecting the battery's lifespan and performance.

Peng *et al.* have reported that these three issues including dendrite growth, corrosion, and HERs are interrelated and can be mitigated using techniques such as interfacial engineering. One effective strategy is to modify the zinc metal anode directly or indirectly by constructing a protective layer at the electrolyte–anode interface. This multifunctional protective layer prevents erosion of the anode by the electrolyte, improves ion transfer kinetics by guiding zinc ions, and controls the volume expansion of the anodes, which helps maintain its shape and performance.^19–21^

## 3. Zinc anode

The anode plays a vital role in battery technology, serving as the starting point for energy storage. In AZIBs, the anode typically composed of zinc metal or foil, is responsible for releasing zinc ions during discharge and reabsorbing them during charging. Often referred to as the “engine room” of the battery, the performance of the anode directly influences the capacity of the battery, lifespan, and overall safety. Despite the advantages of zinc, the anode faces several challenges that have drawn considerable attention from researchers seeking effective solutions. One of the most critical issues is dendrite formation, which the growth of tiny, needle-like zinc structures that occur as zinc ions unevenly on the anode surface during charging. Over time, these dendrites can penetrate the internal layers of the battery, leading to short circuits, sudden failure, and in extreme cases, fires. This phenomenon raises serious concerns, especially given the reputation of AZIBs as a safe and stable energy storage technology.^22,23^ Another major challenge is the hydrogen evolution reaction (HER). This occurs when zinc reacts with water in the aqueous electrolyte, producing hydrogen gas. HER not only wastes energy and generates internal pressure but also reduces the overall efficiency and structural stability of the battery.^24^ Corrosion and surface passivation further undermine anode performance. Because zinc is naturally reactive, it corrodes in water-based electrolytes, leading to the formation of inactive by-products on the anode surface. These by-products hinder the transport of zinc ions, significantly reducing the performance of the battery over time.^25^ Despite these technical hurdles, the importance of the zinc anode in AZIB development cannot be overstated. It holds the key to realizing the high capacity, safety, and affordability that make AZIBs a promising candidate for sustainable energy storage.^26^ As a result, researchers are exploring innovative strategies such as protective coatings, artificial interface layers, electrolyte additives, and 3D-structured anodes. These approaches aim to suppress dendrite growth, minimize corrosion, and promote uniform zinc deposition.^27^ Ultimately, for AZIBs to become viable power sources for homes, electric vehicles, and grid-scale storage, the zinc anode must be made durable, stable, and efficient. These improvements are crucial to unlocking the full potential of this sustainable battery technology. However, for the purpose of this review, the focus will remain on the cathode material used in AZIBs.

## 4. Cathode materials

In addition to the challenges associated with the anode, the cathode is an essential component in developing high-performance battery technology, also faces several significant issues.

Three principal types of materials are considered for use as cathodes in AZIBs or rechargeable AZIBSs: manganese-based oxides, vanadium-based oxides, and Prussian blue and its analogues. Each of these traditional materials presents unique challenges due to their intrinsic properties.

### 4.1. Common challenges

*Small interlayer spacing*: limited interlayer spacing can impede the movement of zinc ions, affecting the efficiency of ion insertion and extraction.

*Strong electrostatic interactions*: zinc ions interact strongly with the crystal structures of the cathode materials, which can hinder ion mobility and reduce overall performance.

**Fig. 1:**
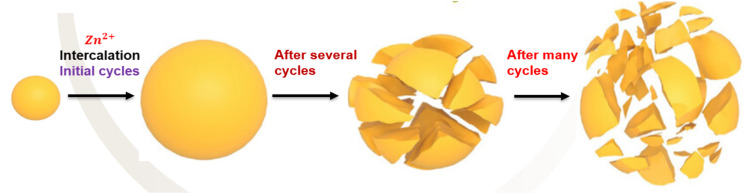
Schematic diagram illustrating cathode volume expansion and dissolution of active materials dissolution cycling.

*Volume expansion*: Fig. 1 highlights how the volume expansion of cathode materials during charge–discharge cycles can result in structural instability and performance degradation.^28^

*Active material dissolution*: dissolution of active materials into the electrolyte can result in decreased capacity and performance of the battery.

These challenges contribute to slow diffusion kinetics, which can hinder the insertion and extraction of zinc ions in the cathode. Consequently, this affects the overall capacity, multiplicity, and cycling performance of AZIBs.^29^

### 4.2. Vanadium-based oxide materials

Vanadium oxide (VO) materials have been investigated as cathodes for rechargeable zinc-ion batteries. However, these materials often suffer from instability during the intercalation process, especially in weakly acidic electrolytes.^30^ During Zn^2+^ cation intercalation, the bonding energy between vanadium and oxygen decreases due to competition between V–O and Zn–O bonds, which reduces thermodynamic stability.^31^ Furthermore, the repeated intercalation of Zn^2+^ cations cause the lattice spacing to expand and contract, leading to structural collapse of the cathode. This instability, along with the lattice deformation during phase transformations, reduces the number of active reaction sites for Zn^2+^ and slows down Zn^2+^ cation transport kinetics.^32^ Additionally, the layered structures of V–O materials often exhibit weak van der Waals forces, resulting in significant structural degradation and posing challenges for designing high-performance battery systems.^33^

#### 4.2.1. Vanadium cathode materials: structure, versatility, and potential

Vanadium oxides materials, a group of transition metal oxides, are considered among the most promising classes of cathode materials in the field of energy storage, particularly for AZIBs. This promise originates from their intrinsic multivalency, structural diversity, and ability to accommodate Zn^2+^ ions reversibly. Vanadium pentoxide (V_2_O_5_), in particular, benefits from multiple stable oxidation states (V^5+^, V^4+^, V^3+^), a rich polymorphism, including orthorhombic V_2_O_5_, hydrated bilayer V_2_O_5_·*n*H_2_O, V_6_O_13_, as well as V_3_O_7_·H_2_O, and an open-layered structure that facilitates ion diffusion. These features contribute to a high theoretical specific capacity >400 mAh g^−1^, broad operating voltage window, and strong redox reversibility.^34^ However, despite these favourable characteristics, vanadium oxide materials, particularly V_2_O_5_, face challenges related to low intrinsic electronic conductivity and structural instability during extended cycling. These limitations can lead to poor rate capability and rapid capacity decay (capacity fading) under practical operating conditions. Recent investigations focused on tackling these bottlenecks through techniques such as nanostructuring, defect engineering, interlayer expansion, and polymer intercalation.^35^

A critical feature of vanadium oxide materials is the flexibility of their VO_*x*_ polyhedral, which allows for reversible coordination changes during ion intercalation/deintercalation, contributing to their structural adaptability.^36^ This flexibility facilitates the stabilization of metastable phases and enables reversible Zn^2+^ storage through mechanisms such as solid-solution reactions and phase transitions. The ability of vanadium-based materials to accommodate a wide variety of intercalating species also leads to a diverse family of derivatives, including vanadium nitrides, carbides, sulfides, phosphates, and metal vanadates, that have been explodes as advanced cathode alternatives.^37^ Among these, V_2_O_5_ remains the most extensively studied due to its relatively straightforward synthesis, environmental benignity, and high operating voltage. Notably, V_2_O_5_ has demonstrated promising performance enhancements when modified with conductive polymers, heteroatom dopants, or pillared by metal ions to expand the interlayer spacing and buffer structural degradation.^38,39^ Recent insights from *operando* spectroscopy and DFT calculations suggest that rationally tuning the electronic structure and Zn^2+^ diffusion pathways of V_2_O_5_ can result in transformative improvements in rate performance and cycling life.^40^

**Fig. 2:**
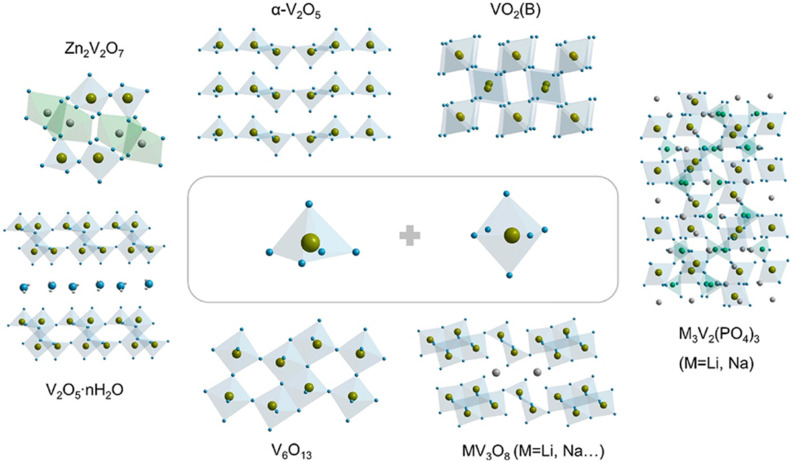
Crystal structure of typical vanadium oxides: orthorhombic α-V_2_O_5_; monoclinic VO_2_(B), V_2_O_5_·*n*H_2_O, and MV_3_O_8_ (M = Li, Na, K, …); trigonal V_6_O_13_ and M_3_V_2_(PO_4_)_3_ (M = Li, Na, K, …); and triclinic Zn_2_V_2_O_7_.^18^

Fig. 2 illustrates various vanadium oxide structures commonly employed as cathode materials, demonstrating the versatility and structural richness of this material family. As the field advances, an increasingly rational approach to material design, grounded in mechanistic understanding and supported by computational modelling is expected to bridge the gap between laboratory discovery and real-world deployment.

Vanadium oxide materials, a group of transition metal oxides, are considered promising candidates for cathode materials in the energy sector due to their intrinsic characteristics. These include various oxidation states, diverse crystalline structures, excellent theoretical specific capacity, and a broad output voltage range.^41^ Despite these advantages, vanadium oxides often struggle with issues related to conductivity and cycling stability, which limit their effectiveness as cathode materials.^42^ The use of vanadium compounds as cathode materials has gained traction due to several key factors. These include their multiple valence states and the flexible nature of V–O polyhedral bonds, which enable partial electroneutrality through variations in the oxidation state of vanadium, leading to stable crystalline structures.^41^ Additionally, the ability of vanadium to accommodate various anions and cations results in a wide range of compounds utilized across different scientific fields. In renewable energy storage systems, especially in the design and development of AZIBs, various vanadium-based compounds are employed.^43^ These include vanadium oxides, metal vanadates, vanadium phosphates, vanadium carbides, vanadium nitrides, and vanadium sulfides. Vanadium oxides are particularly sought after due to their multiple valence states, which contribute to specific structural characteristics and enhance the storage capacity by accommodating and releasing Zn cations in various ways. The most studied vanadium oxides are VO_2_, V_2_O_3_, V_2_O_5_, bilayer V_2_O_5_·*n*H_2_O, V_3_O_7_·H_2_O, and V_6_O_13_.^43,44^Fig. 2 showcases various structural configurations of vanadium-based oxide materials employed as cathodes.

This review will specifically focus on V_2_O_5_. However, the Table 1 below outlines some key vanadium-based materials used as cathodes in AZIBs, along with their primary characteristics. Each material has been evaluated for properties such as capacity, stability, and advantages in the context of AZIB.

**Table 1:** Overview of various vanadium oxide materials: structural characteristics, electrochemical performance, advantages and challenges

Material	Structural characteristics	Stability	Electrochemical performance (capacity)	Voltage window (V)	Current densities (A g^−1^)	Interlayer spacing (A)	Advantages	Challenges	References
V_2_O_5_	Orthorhombic layered	Moderate	∼294 mAh g^−1^	0.2–1.6	0.2–2.0	∼4.4	High theoretical capacity; simple synthesis	Structural collapse, poor cycling	45 and 46
Hydrated V_2_O_5_·*n*H_2_O	Layered with intercalated water	High	∼370 mAh g^−1^	0.2–1.6	0.1–1.0	∼11.9	Enlarged interlayer spacing for fast Zn^2+^ diffusion	Water loss, instability over time	7, 47 and 48
VO_2_(B)	Monoclinic	Good	∼276 mAh g^−1^	0.2–1.4	0.1–1.5	∼2.9	Fast kinetics, tunnel-type channels	Moderate capacity	49 and 50
V_6_O_13_	Mixed valence, layered	Moderate	∼280 mAh g^−1^	0.3–1.4	0.1–1.0	∼4.7	Mixed V^4+^/V^5+^ for better conductivity	Phase transition during cycling	51
δ-V_2_O_5_	Expanded layered phase	Good	∼350 mAh g^−1^	0.2–1.6	0.1–2.0	∼10.8	Enhanced ion diffusion	Difficult synthesis phase purity	11 and 52
Zn_*x*_V_2_O_5_·*n*H_2_O (pre-intercalated)	Layered with Zn^2+^ and H_2_O	High	∼350–400 mAh g^−1^	0.2–1.6	0.1–2.0	∼13.1	Stabilized structure, fast Zn^2+^ insertion/extraction	Complexity in controlling stoichiometry	53 and 54
V_2_O_5_@carbon/polymer composites	Composite/hybrid	Very high	∼400 mAh g^−1^	0.2–1.6	Up to 5.0	>10	Improved conductivity, flexibility	Fabrication complexity	55

#### 4.2.2. Vanadium pentoxide (V_2_O_5_)

**Fig. 3:**
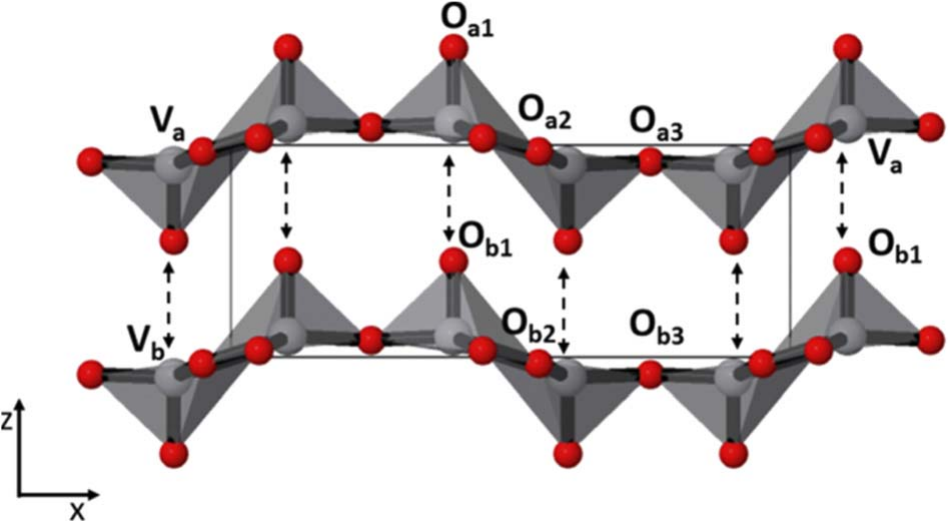
Schematic structure of V_2_O_5_ along *c* axis.^62^

Vanadium pentoxide (V_2_O_5_) is a transition metal oxide that features an orthorhombic crystal system with a space group of *Pmmm*. It is characterized by a typical two-dimensional layered structure. Structurally, vanadium pentoxide consists of vanadium atoms centrally positioned and bonded to five oxygen atoms, forming a square pyramidal [VO_5_] unit. These square pyramids [VO_5_] are arranged in layers by sharing edges (or corners), creating a cohesive and layered framework.^56^ According to the investigation conducted by Zhang *et al.*,^57^ vanadium pentoxide (V_2_O_5_) features van der Waals forces and hydrogen bonds that link adjacent layers together. This layered structure is crucial for its use in energy storage systems, as it facilitates the easy intercalation and deintercalation of Zn cations during charge–discharge cycles. Additionally, the transfer of electrons to the current collector occurs within a single charge–discharge cycle due to the multiple oxidation states of vanadium, ranging from +2 to +5. This, combined with the high theoretical capacity of V_2_O_5_, makes it a promising material for energy storage applications.^58,59^ Moreover, vanadium pentoxide (V_2_O_5_) is considered a promising ion host in energy storage systems due to its strong faradaic activity, low cost, eco-friendliness, high capacitance, and high energy density. Its consistent crystal structure and natural abundance further enhance its appeal as a material for energy storage applications.^60^ The exploration of V_2_O_5_ for energy conversion, nano storage devices, and specifically in the domain of AZIBs is driven by several key benefits: its ability to accommodate molecules and ions, exceptional catalytic activity with a high number of active sites, and strong electron–electron correlation.^61^Fig. 3 presents the schematic representation of the V_2_O_5_ structure along the *c*-axis, highlighting its structural arrangement.

**Fig. 4:**
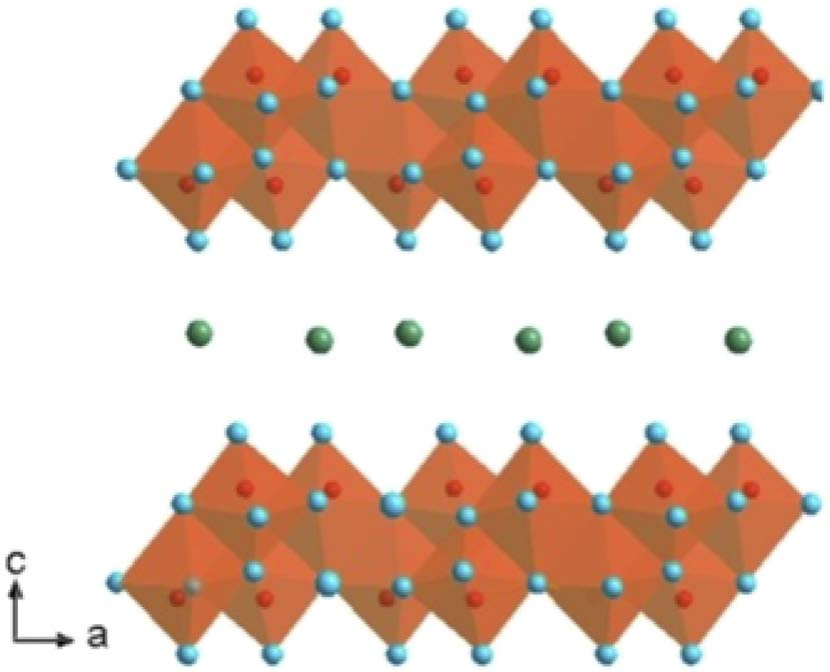
Vanadium pentoxide 3D structure.^269^

V_2_O_5_ has garnered attention in energy storage research due to its layered structure, which facilitates easy intercalation and deintercalation of ions during charge–discharge cycles. Additionally, its multiple oxidation states and significant storage capacity contribute to efficient electron transfer to the current collector through a straightforward charge–discharge process.^58,59^Fig. 4 illustrates the three-dimensional layered structure of V_2_O_5_, emphasizing its unique arrangement.

The V_2_O_5_ possesses a strong faradaic activity, consistent crystal structure, and natural abundance occurrence which are presented as advantages to be considered in the selection of electrode materials.^60^ V_2_O_5_ exhibits various morphologies depending on the technology used, including nanorods, nanowires, nanotubes, nanoribbons, and nanoparticles. Among these, V_2_O_5_ nanorod electrodes stand out for their exceptional electrochemical properties, as studies have shown they outperform other V_2_O_5_ morphologies.^63,64^ To produce these electrode materials, various synthesis techniques have been employed to prepare different types of V_2_O_5_. These methods include:

• Sol–gel hydrothermal/solvothermal method

• Template approach

• Electrospinning method

• Atomic layer deposition method

• Electrodeposition method

Each technique offers distinct advantages in tailoring the morphology and properties of V_2_O_5_ for specific applications.^65^

The various technologies used to synthesize and prepare V_2_O_5_ will be detailed in a later section of this review. The next section will focus on the crystallographic structure and phase transitions of V_2_O_5_.

#### 4.2.3. Crystal structure properties and phase transitions

**Fig. 5:**
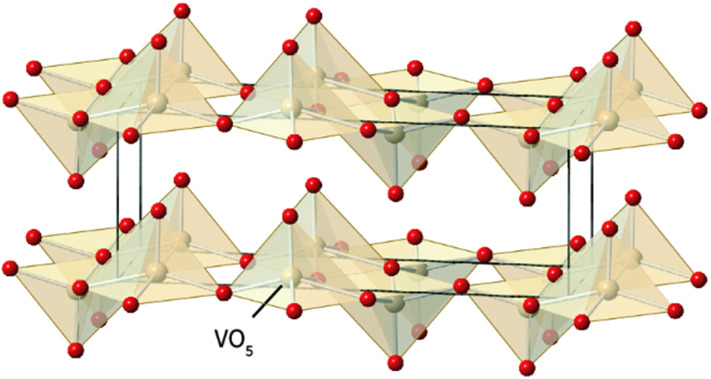
The structure of vanadium oxide consists of interconnected VO_5_ square pyramidal units.^68^

**Fig. 6:**
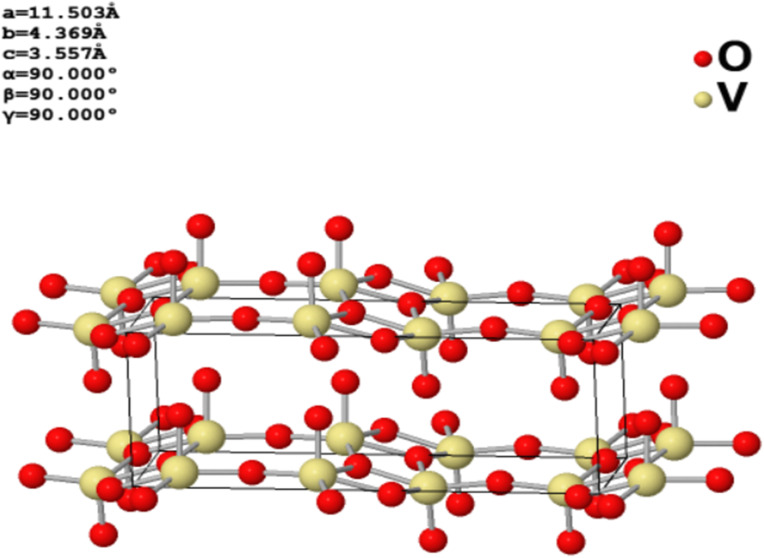
The structure of vanadium pentoxide (V_2_O_5_) features a layered arrangement of polyhedral units, primarily composed of distorted VO_5_ square pyramids and VO_6_ octahedra.^68^

Vanadium oxide (V–O) compounds are crucial materials currently being explored for the design of cathodes in AZIBSs due to their notable advantages, including stability and structural diversity. The V–O polyhedral can adopt various crystalline structures, such as tetrahedra, trigonal bipyramids, square pyramids, distorted octahedra, and regular octahedra. This structural flexibility arises from the different oxidation states of vanadium, which allows for a range of coordination environments.^66,67^ According to Ming *et al.*,^17^ the various oxidation states of vanadium lead to a diverse range of vanadium oxides, each with different polyhedral structures that can facilitate reversible Zn^2+^ intercalation and deintercalation. These structures typically share common corners and/or edges, facilitating the insertion and removal of zinc ions, as illustrated in the Fig. 5 and 6 below.

As previously mentioned, vanadium oxides can span a range of oxidation states from V^2+^ to V^5+^. Specifically, in vanadium pentoxide (V_2_O_5_), the oxidation state of vanadium typically ranges from V^5+^ to V^4+^. Some studies suggest that vanadium can even be reduced further to V^3+^ under certain conditions.^69^

This work investigates vanadium pentoxide (V_2_O_5_), which is composed of VO_5_ square pyramidal units and VO_6_ octahedral units. Structurally, V_2_O_5_ is described as a layered orthorhombic compound. The crystal structure of V_2_O_5_ consists of alternating of distorted VO_5_ square pyramids and VO_6_ octahedra. These coordination polyhedral are connected through corner-and edge-sharing, forming extended layers along the *ab*-plane. Typically, each VO_6_ octahedron shares one to two edges and one corner with adjacent VO_5_ pyramids, resulting in a characteristic “up–up” and “down–down,” stacking motif along the *c*-axis. The V–O bond lengths within these polyhedral range from approximatively 1.6 to 2.0 Å, reflecting the asymmetric coordination environment and mixed-valence nature of vanadium ions.^70^ This quantitative description highlights the pronounced distortion and mixed coordination environment, which are important to the electrochemical behaviour of the material.^70^ According to the investigation conducted by Le *et al.*,^69^ despite the multiple oxidation states observed in V_2_O_5_, its structural shape remains unchanged. The layered structure and the presence of various oxidation states are crucial for using V_2_O_5_ as a cathode material, particularly in the design of AZIBs. These characteristics enable efficient metal intercalation and deintercalation during charge–discharge cycles. Additionally, they simplify the storage mechanisms in V_2_O_5_ compared to the more complex mechanisms observed with manganese oxide cathode materials.^71,72^

**Fig. 7:**
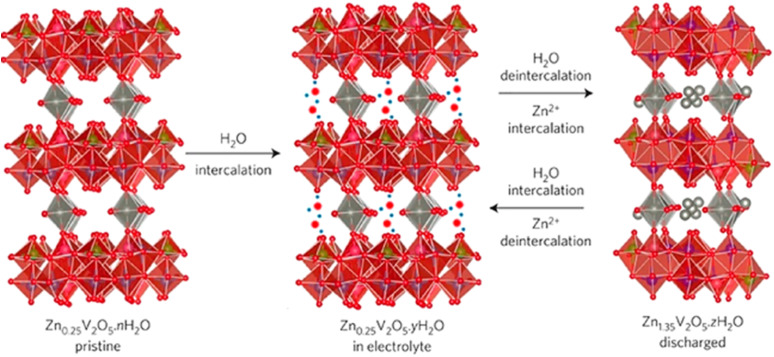
Schematic of the water intercalation into Zn_0.25_V_2_O_5_·*n*H_2_O upon being immersed in aqueous electrolytes and the simultaneous Zn^2+^ intercalation–deintercalation and water deintercalation upon cycling.^79^

In addition to the orthorhombic V_2_O_5_ structure, there are two other notable forms of V_2_O_5_: the amorphous structure and the hydrated V_2_O_5_ structure. The following sections will explore the crystallographic details of amorphous V_2_O_5_ and hydrated V_2_O_5_. Amorphous V_2_O_5_ is a prominent material in energy storage systems, particularly in the design of AZIBs. Recent advancements have introduced a 2D heterostructure for amorphous V_2_O_5_, created by interchanging pairs of distinct 2D nanosheets. This approach offers enhanced synergy compared to the traditional orthorhombic V_2_O_5_, which is typically used in composites involving hybridized 2D materials with nanoparticles or mixtures of different 2D materials. Unlike traditional composites, where poor interfacial contact and phase separation often limit synergistic effects, the engineered 2D heterostructure of amorphous V_2_O_5_ exhibits markedly enhanced electrochemical properties. This structure offers a significantly shortened Zn^2+^ diffusion path, typically less than 10 nm, an increased density of electrochemically active sites due to its disordered structure, and improved electrical conductivity, often enhanced by several orders of magnitude when combined with conductive additives or substrates from ∼10^−2^ to 1 S cm^−1^.^73,74^ These enhancements stem from the interface between nanoscale domains and the lack of long-range crystallinity, which minimizes lattice strain and facilitates rapid ion transport.^75,76^ Hydrated V_2_O_5_ is another important form used in energy storage systems, particularly in the design of AZIBSs. Its crystal structure features V_2_O_5_ double chains, which consist of edge-shared VO_6_ octahedra. Water molecules are interspersed between these adjacent layers, playing a crucial role in the structure's stability and performance.^77^ The water molecules in the hydrated V_2_O_5_ structure play a crucial role in enhancing the performance of AZIBSs. They shield the effective charge of Zn^2+^ ions and expand the V_2_O_5_ layers, which helps to minimize the electrostatic interactions between Zn^2+^ cations and the host material. Additionally, this hydration contributes to the rapid transport of Zn^2+^ ions while maintaining the structural integrity of the V_2_O_5_, ensuring efficient battery performance without deformation of the host material.^12,78^Fig. 7 illustrates the pre-intercalation of water into pristine V_2_O_5_ and its impact on the interlayer spacing, showcasing the resulting structural modifications.

During charge–discharge cycles, V_2_O_5_ undergoes phase transitions, particularly during the discharge period. In this phase, Zn^2+^ cations and water molecules co-intercalate into the layered structure of V_2_O_5_, resulting in the formation of a new layered phase, Zn_*x*_V_2_O_5_·*n*H_2_O. This transformation occurs during discharge and the newly formed structure reverts to V_2_O_5_ during charging. Additionally, the repeated insertion and extraction of Zn^2+^ cations induce morphological changes in V_2_O_5_, evolving from thin and smooth nanosheets to a porous structure over extended cycling. According to investigations conducted by Jia *et al.*,^18^ these phase transitions and morphological transformations impact the performance and stability of the material in energy storage applications, the newly formed porous structure increases the surface area of V_2_O_5_ from 13.6 to 118.4 m^2^ per gram. This expansion significantly enhances the number of active sites available for Zn^2+^ cation storage, thereby boosting the capacity during the initial phase of charging cycles.

#### 4.2.4. Electrochemical properties

This paragraph will review the electrochemical properties of V_2_O_5_, including redox reactions, voltage profiles, and capacity retention. The crystal structure of vanadium oxide-based materials plays a significant role in these properties. The diverse structures of vanadium oxides contribute to their high theoretical specific capacity and wide output voltage range. As a result, vanadium oxide-based materials are highly considered for use as cation host materials in the design of electrical energy storage systems.^42^ Despite these advantages, vanadium oxide-based materials are often characterized by weak electrical conductivity and limited cycling properties.^80^

This paper will later propose strategies and techniques to address the issues of weak electrical conductivity and limited cycling properties. The next section will explore the reaction storage mechanisms occurring in vanadium pentoxide.

##### 4.2.4.1. Storage reaction mechanism

Vanadium pentoxide (V_2_O_5_) has a unique layered structure that enhances its electrochemical performance, particularly during the intercalation and de-intercalation of Zn^2+^ ions. In AZIBSs, the storage reaction mechanism involves two key processes:

(1) *Insertion*: during the charging process, Zn^2+^ cations are inserted into the V_2_O_5_ lattice.

(2) *Extraction*: during discharge, Zn^2+^ cations are extracted from the V_2_O_5_ lattice.

**Fig. 8:**
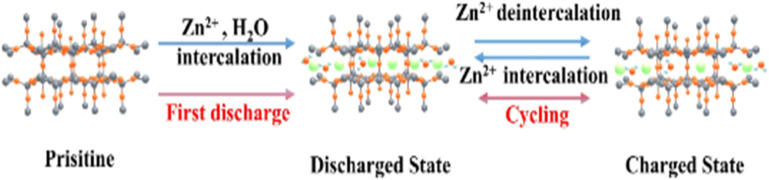
Schematic illustration of Zn^2+^ and water co-intercalation into V_2_O_5_.^81^

**Fig. 9:**
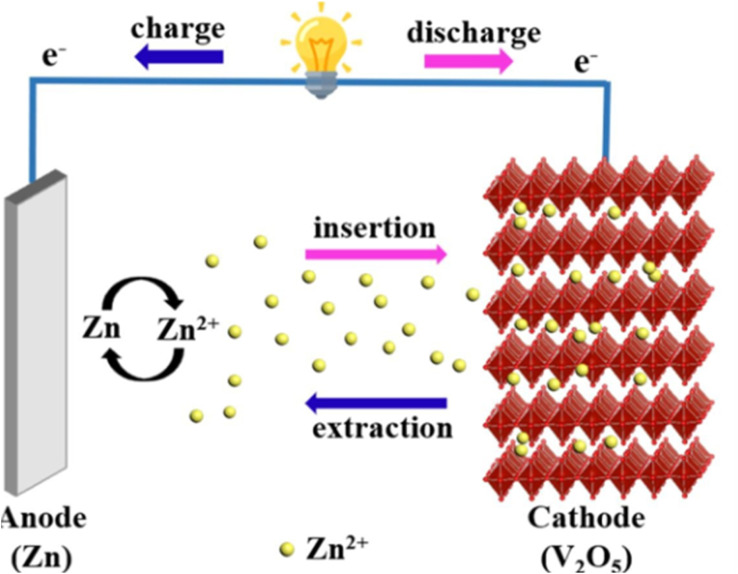
Schematic illustration of the electrochemical behaviour of Zn/V_2_O_5_ system during operation.^82^

This intercalation and de-intercalation process is crucial for the battery's operation and performance. The Fig. 8 and 9 below illustrate the intercalation and deintercalation processes that occur during battery operation.

##### 4.2.4.2. Reactions

The discharge and charge processes in V_2_O_5_-based AZIBSs are crucial for understanding their electrochemical behaviour.

• *Discharge process*: during discharge, Zn^2+^ cations from the aqueous electrolyte intercalate into the V_2_O_5_ structure, reducing the vanadium ions. The reaction is:V_2_O_5_ + Zn^2+^ + 2e^−^ → ZnV_2_O_5_

• *Charge process*: during charging, Zn^2+^ cations are extracted from the V_2_O_5_ structure, oxidizing the vanadium ions back to their original state. The reaction is:ZnV_2_O_5_ → V_2_O_5_ + Zn^2+^ + 2e^−^

Mechanistically, the interlayer spacing within the layered V_2_O_5_ structure plays a critical role. It accommodates Zn^2+^ ions through diffusion during intercalation. As Zn^2+^ ions are inserted, V^5+^ ions are reduced to V^4+^ or V^3+^ to balance the charge. This insertion and extraction cause structural variations in the lattice, leading to expansion or contraction. These structural changes can impact the electrochemical performance of the battery, as weak structural stability and poor cycling performance are often observed.^83^ According to Tay *et al.*,^83^ when Zn^2+^ cations intercalate into the V_2_O_5_ structure, the cathode expands due to the increased interlayer spacing required to accommodate the larger Zn^2+^ ions. This expansion also involves a solvation shell of water molecules surrounding the cations. The intercalation of Zn^2+^ cations into the interlayer spacing is a crucial process for charge storage during the insertion process. However, it's important to note that changes in the pH of the electrolyte during discharge can lead to additional types of intercalations that generate by-products. These by-products can affect the electrochemical performance and stability of the cathode. Additionally, this type of intercalation, which occurs in response to varying electrolyte pH, is not observed in all oxide compounds but is specific to certain types. This variability in behaviour underscores the importance of carefully managing electrolyte conditions to optimize battery performance and longevity.^22,27^

The next paragraph will explore the different types of intercalation processes and their benefits in the operation of rechargeable aqueous zinc-ion batteries.

##### 4.2.4.3. Intercalations

**Fig. 10:**
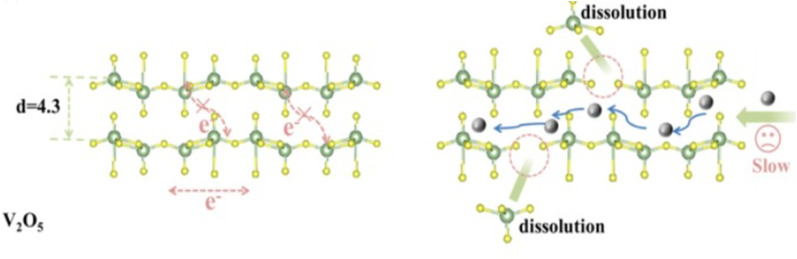
The intercalation process can lead to the dissolution of the V_2_O_5_ cathode, primarily due to the large size of the intercalating cations [Zn^2+^].^87^

Intercalation is a key process in the operation of rechargeable aqueous zinc-ion batteries (AZIBSs), and it can be classified into two main types: the intercalation of Zn^2+^ cations and H^+^ protons. During the discharge process, Zn^2+^ cations are intercalated into the vanadium pentoxide (V_2_O_5_) lattice, enhancing charge storage and overall battery performance. Additionally, H^+^ protons also intercalate into the lattice spacing of the cathode, particularly in oxide–material cathodes. Unlike Zn^2+^ cations, H^+^ intercalation results in changes to the electrolyte pH, which can lead to the formation of by-products. Intentional intercalation involves inserting foreign guest species into V_2_O_5_ to further improve the cathode's electrochemical performance. This process can enhance battery performance by optimizing charge storage.^22,27^ The intercalation of Zn^2+^ cations cause the expansion of the cathode's interlayer spacing due to their larger size compared to the interlayer spacing of vanadium-oxide cathodes.^84^ This expansion is also influenced by the solvation shell of water molecules surrounding the Zn^2+^ cations, which is approximately 2.1 Å compared to 0.88 Å for unsolved Zn^2+^ with a coordination number of six. In contrast, non-oxide cathode materials can exhibit lattice contraction (≤0.5 Å) due to interactions between the bivalent Zn^2+^ ions and electron-rich centers in the materials.^46,85,86^ The Fig. 10 below illustrates the intercalation process occurring within the V_2_O_5_ cathode, highlighting the sluggish kinetics of cations during intercalation process.

**Fig. 11:**
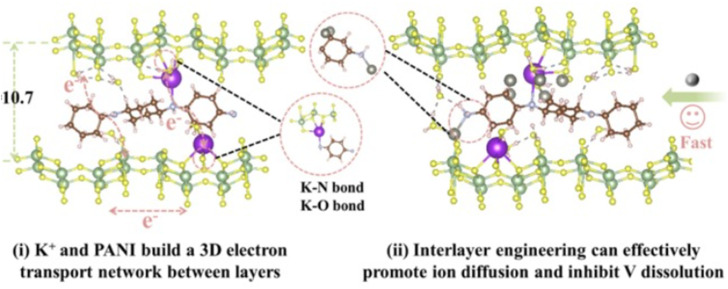
Pre-intercalation technique using to enhance the electrochemical performance of V_2_O_5_ by expanding the interlayer spacing and improving ion diffusion pathways.^87^

The intercalation of Zn^2+^ cations plays a vital role in the performance of AZIBSs. This process induces an expansion in the interlayer spacing, which stabilizes the cathode and facilitates efficient ion de-intercalation. Research by Zhang *et al.* demonstrates that as Zn^2+^ cations enter the lattice structure of V_2_O_5_ during battery operation, they form a stable, low-crystallinity phase. This reduced crystallinity lowers kinetic barriers, enhancing charge storage in subsequent cycles and improving battery performance by enabling better Zn^2+^ cation diffusion and increasing the number of active sites.^46^ It is important to note that energy barriers significantly influence Zn^2+^ cation diffusion.^46,86,88^ Reducing these barriers and increasing the number of active sites in the cathode material improve ion transfer kinetics and the reversibility of Zn^2+^ cation intercalation during discharge, thereby further enhancing battery performance. To optimize battery performance, the introduction of foreign species into the cathode has been proposed. Pre-intercalation of foreign species, including alkali metals^84^ and small molecules,^86^ aims to expand the interlayer spacing and stabilize the cathode material for operation over numerous charge–discharge cycles.^27^ For instance, research by Du *et al.* demonstrated that incorporating cations such as Mn, Fe, Co, Ni, Ca, and K into the V_8_O_20_ cathode enhanced its capacity and performance, with Mn pre-intercalation leading to significant capacity retention over 1000 cycles.^89^ Additionally, studies have explored the pre-intercalation of molecules like ammonia and organic nitrogen materials to further enhance the electrochemical performance of cathodes.^86,90–92^ To improve the electrochemical performance of V_2_O_5_ cathode materials, several strategies will be reviewed later, including the intercalation of various guest species and polymers. The intercalation of Zn^2+^ cations into the interlayer spacing of V_2_O_5_ can significantly enhance battery performance. This process leads to the formation of a stable, low-crystallinity structure, which reduces kinetic barriers and prevents additional Zn^2+^ cations from intercalating. As a result, the cathode's capacity to store charge during each cycle is improved. This stability and reduction in energy barriers are crucial for efficient Zn^2+^ ion diffusion within the host material.^84,86,91^ The Fig. 11 below demonstrates how pre-intercalation enhances electrochemical performance and increases interlayer in the material.

##### 4.2.4.4. Mechanism of Zn^2+^ intercalation in V_2_O_5_: interaction sites and binding forces

Vanadium pentoxide (V_2_O_5_), a layered transition metal oxide, is considered one of the most promising cathode materials for AZIBs. This is due to its high theoretical capacity (∼294 mAh g^−1^), multivalent redox behaviour, and an open-layered structure that readily accommodates Zn^2+^ ion intercalation.^34^ These intercalated ions not only influence the crystallographic properties of V_2_O_5_ but also interact with the interact with the intrinsic physicochemical characteristics of Zn^2+^ itself.^35,93^

##### 4.2.4.5. Crystallographic location of Zn^2+^ ions

Structurally, vanadium pentoxide α-V_2_O_5_ adopts an orthorhombic crystal system (space group *Pmmn*), composed of VO_5_ square pyramids connected *via* edge and corner-sharing to form two-dimensional layers aligned along the *b*-axis. These layers are held together by weak van der Waals forces, creating interlayer voids that serve as pathways and storage sites for Zn^2+^ ions during battery discharge. When intercalated, Zn^2+^ ions occupy these interstitial spaces, where they are coordinated by multiple oxygen atoms from nearby vanadium centres.^34^

Advanced structural characterization methods, such as X-ray diffraction (XRD), neutron diffraction, and pair distribution function (PDF) analysis, have revealed that Zn^2+^ ions tend to occupy octahedral or distorted trigonal prismatic sites, typically surrounded by five to six oxygen atoms.^93^ These coordinating oxygen atoms, including both bridging (O_br) and terminal (O_t) types derived from V=O bonds, form a quasi-stable local environment capable of accommodating the high charge density of Zn^2+^ ions.^35^ Additionally, density functional theory (DFT) calculations have identified preferred crystallographic positions for Zn^2+^ ions, notably around the fractional coordinates (0.5, 0.25, 0.5), where they achieve substantial stabilization energy.^35^

##### 4.2.4.6. Types of interaction forces governing Zn^2+^ binding

The reversible intercalation of Zn^2+^ ions into the V_2_O_5_ host structure is stabilized through a combination of electrostatic interactions, coordination bonding, hydrogen bonding, and van der Waals forces. The dominant intercalation arises from the strong electronic (coulombic) attraction between the divalent Zn^2+^ ions and the negatively charged O^2−^ anions within the V_2_O_5_ lattice. This electrostatic force energetically favours the insertion of Zn^2+^ ions into the interlayer spacing (voids).^94^ In addition to electrostatic stabilization, coordination bonding plays a significant role.^95^ Upon intercalation, Zn^2+^ ions interact with surrounding oxygen atoms to form distorted octahedral geometries, where partial orbital overlap between Zn^2+^ 3d and O 2p orbitals contributes to further stabilization, surpassing the effects of pure ionic interactions.^94^ These ligand field effects are also important in determining the thermodynamic preference for specific interaction sites within the host structure. In aqueous electrolytes, Zn^2+^ ions typically exist in a hydrated form, most commonly as [Zn(H_2_O)_6_]^2+^.^94,96^ During co-intercalation with water molecules, hydrogen bonding and ion–dipole interactions occur between the hydrated Zn^2+^ complex and the cathode lattice.^97^ These interactions not only reduce the local lattice strain but also promote the formation of a quasi-hydrated Zn–O coordination network, enhancing the structural stability of the host material (cathode V_2_O_5_) during cycling.^98^ Furthermore, although weaker in nature, van der Waals forces between adjacent V–O layers contribute by accommodating volumetric expansion induced by Zn^2+^ insertion and mitigating structural deformation, thereby supporting the long-term cycling stability of the electrode.^99^

##### 4.2.4.7. Voltage profiles

The exploration of V_2_O_5_ as cathode material for high-performance AZIBs is driven by its layered crystal structure, high theoretical capacity, and ability to undergo multivalent redox transitions, enabling multiple electron transfers during cycling. A comprehensive understanding of its voltage profiles offers crucial insights into the electrochemical mechanisms, phase transitions, and structural dynamics that govern battery performance During discharge, Zn^2+^ ions migrate from the anode into the V_2_O_5_ lattice, reducing vanadium cations from V^5+^ to V^4+^ and in some case to V^3+^. Initially, this intercalation follows a solid-solution mechanism, reflected by sloping voltage profile between 1.6 and 1.4 V *versus* Zn/Zn^2+^.^100,101^ This followed by appearance of distinct voltage plateaus, associated with two-phase reactions and Zn^2+^ insertion into specific crystallographic sites. These phase transitions corresponded to measurable lattice expansions, particularly along the *b*-axis, as confirmed by *in situ* XRD and *operando* Raman spectroscopy.^102,103^ As deeper Zn^2+^ intercalation occurs, typically below 1.2 V, the process becomes increasingly energetically demanding, producing a broader sloping region down to ∼1.0 V, indicative of mixed-phase formation such as Zn_*x*_V_2_O_5_.^104^ The charging process reverses these structural changes, with Zn^2+^ ions de-intercalating from the V_2_O_5_ cathode's layers and vanadium cations oxidized back to V^5+^. This process is accompanied by voltage plateaus in the 1.4–1.6 V range and a final rise to ∼1.8 V as Zn^2+^ is fully extracted. However, full structural restoration is not always achieved due to cumulative lattice strain or residual Zn^2+^ trapped in the structure, which contributes to voltage hysteresis and capacity fading over prolonged cycling.^6,79,105,106^

Electrolyte composition significantly influences these voltages behaviours. Mildly acidic or near-neutral ZnSO_4_ and Zn(CF_3_SO_3_)_2_ electrolytes help suppress side reactions like hydrogen evolution, stabilizing both the voltage profile and the V_2_O_5_ lattice during cycling. In contrast, high-pH environments often promote dissolution and irreversible changes in the V_2_O_5_ structure. High-concentration “water-in-salt” electrolytes have also shown promise in widening the stable operating voltage window and enhancing cathode reversibility.^107^

**Fig. 12:**
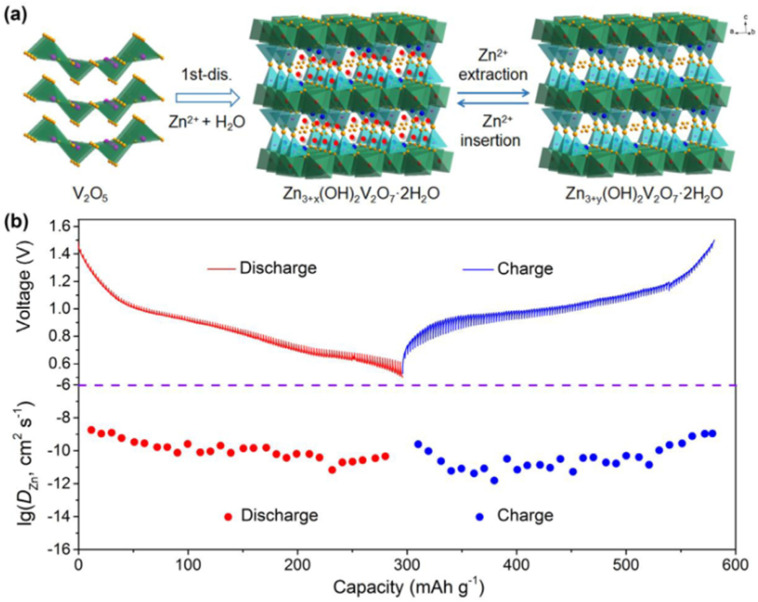
(a) Co-intercalation reaction mechanism involving Zn^2+^ ions and water molecules, illustrating their simultaneous insertion into V_2_O_5_ structure.^108^ (b) Voltage profile of V_2_O_5_, showing the electrochemical behaviour during charge and discharge cycles.^108^

The typical operating voltage range for V_2_O_5_-based AZIBs falls between 0.3 and 1.6 V, balancing the need for reversible Zn^2+^ intercalation and structural stability. Notably, recent studies have shown that water molecules often co-intercalate with Zn^2+^, softening the lattice and modulating the local bonding environment, which in turn affects both the voltage profile and cycling kinetics.^100,101^ Thus, integrating electrochemical data with crystallographic analysis is essential for a comprehensive understanding of V_2_O_5_ voltage behaviour and its optimization in next-generation AZIB systems. Fig. 12 depicts the co-intercalation of Zn^2+^ and water molecules, along with their impact on the voltage profiles of modified V_2_O_5_ cathode.

###### 4.2.4.7.1. Parameters affecting the voltage profile

This paragraph discusses the key parameters that influence the voltage profile of V_2_O_5_ in AZIBSs, including electrolyte composition, V_2_O_5_ morphology and particle size, as well as structural modifications of V_2_O_5_. The electrolyte, being a critical component of the battery, requires precise tuning of its composition, particularly the pH and Zn^2+^ concentration, as these factors significantly impact the voltage profile. The pH and Zn^2+^ concentration play a pivotal role in ion transfer kinetics during the intercalation and de-intercalation processes, also affect the stability of the V_2_O_5_ host structure. Second, the morphology and particle size of V_2_O_5_ are crucial factors that influence the voltage profile.^109^ Optimizing particle size, especially by employing smaller or nanostructured V_2_O_5_ cathodes with numerous active sites, enhances ion transfer kinetics, resulting in a more pronounced voltage profile with reduced polarization. Lastly, the structure of the V_2_O_5_ cathode itself is another critical parameter that requires modification. However, detailed discussion of the structural modifications is beyond the scope of this paragraph, as a dedicated section will address the various techniques suitable for modifying the cathode structure, thereby improving the stability of the voltage plateaus and enhancing the overall electrochemical performance of the battery.^30^

##### 4.2.4.8. Capacity retention

Capacity retention is a critical metric for evaluating the electrochemical performance of cation host materials in batteries, including cathodes in rechargeable AZIBs.^110^ In the context of designing high-performance AZIBs, the capacity retention of selected cathode materials, such as V_2_O_5_, is particularly significant. Capacity retention is defined as the intrinsic ability of a cathode material to maintain its charge storage capacity after numerous charge–discharge cycles.^111,112^ It serves as an indicator of how well the material has been engineered to sustain its electrochemical performance over an extended period, without undergoing structural degradation.^113^

###### 4.2.4.8.1. Capacity retention of V_2_O_5_ in AZIBSs

**Fig. 13:**
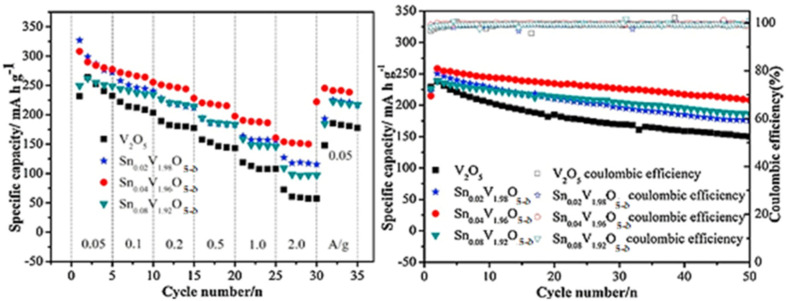
Rate tests and cycling performance of electrodes at 200 mAh g^−1^.^117^

As previously discussed in this review, V_2_O_5_ has been extensively employed as a cathode material in the design of AZIBs due to its intrinsic properties, such as its layered structure, which facilitates the intercalation and de-intercalation of Zn^2+^ cations within the V_2_O_5_ interlayer spacing. The significant interlayer spacing in V_2_O_5_ enhances the mobility of Zn^2+^ cations through the electrolyte during battery operation, resulting in a high initial capacity. Various studies have shown that V_2_O_5_ can deliver an initial capacity in the range of 300–400 mAh g^−1^.^100,114^ However, this capacity range is influenced by the synthesis technique used for V_2_O_5_, the operating conditions (such as temperature and pressure), and any structural modification techniques employed.^115^ During the charge–discharge cycles of AZIBSs, Zn^2+^ cations are inserted into and extracted from the V_2_O_5_ interlayer spacing, leading to several changes within the cathode material. These changes include structural degradation, dissolution of active sites, and the occurrence of side reactions, all of which adversely affect capacity retention over time. However, cathodes that have been carefully fabricated using modification techniques such as pre-intercalation of guest species, nanostructuring, compositional adjustment with materials like polymers, and proactive coatings can achieve capacity retention in the range of 80–90% after several hundred cycles,^115,116^ as displayed in the Fig. 13 below. These aspects will be discussed in detail later in the review.

###### 4.2.4.8.2. Challenges affecting capacity retention of V_2_O_5_

As already mentioned, the V_2_O_5_ cathode undergoes changes during its operation, which are attributed to structural degradation, electrolyte instability, and the dissolution of V_2_O_5_ in the electrolyte.^114,116^

• Structural degradation: repeated insertion and extraction of Zn^2+^ cations can lead to the gradual breakdown of the cathode structure.

• Electrolyte stability and dissolution of V_2_O_5_ in the electrolyte: the stability of the electrolyte in contact with the cathode material is crucial. Degradation of the electrolyte over time or reactions between the V_2_O_5_ cathode and the electrolyte during operation can reduce the number of active sites (*e.g.*, pores) available for electrochemical reactions, leading to diminished capacity retention.^115^

Several strategies have been explored to address the challenges associated with capacity retention. Some of these strategies, such as the pre-intercalation of the guest species, have already been discussed. Additional strategies will be examined, including emerging techniques currently under investigation. Notably, these include the incorporation of polymers and the utilization of chemical functional groups as sources of active materials, offering promising avenues to enhance the performance and stability of V_2_O_5_-based cathodes.

###### 4.2.4.8.3. Performance metrics

Two notable performance metrics related to the capacity retention of V_2_O_5_ cathodes are long-term cycling stability and rate capacity. Various studies have emphasized the importance of implementing proper modification strategies and optimization techniques during the design of V_2_O_5_ cathodes to achieve excellent capacity retention, typically in the range of 80–90% after 1000 cycles, which is indicative of good long-term cycling stability. Additionally, V_2_O_5_-based cathodes exhibit good rate capability, meaning they can maintain a reasonable capacity even at high-charge–discharge rates. However, capacity retention at higher rates may decline more rapidly compared to lower rates due to the increased stress on the material.^100,114^

## 5. Synthesis and characterization techniques

This section provides a detailed analysis of the various techniques used for the synthesis and characterization of V_2_O_5_ cathode material.

### 5.1. Synthesis techniques

Several synthesis techniques are employed in the production of V_2_O_5_; however, this review specifically focuses on three widely utilized methods: sol–gel process, hydrothermal synthesis, and the traditional solid-state reaction technique. These methods are selected due to their distinct advantages in tailoring the material properties of V_2_O_5_ to meet specific application requirements. The sol–gel method offers precise control over material composition and microstructure, making it ideal for producing nanostructured and high-purity V_2_O_5_. Hydrothermal synthesis enables the growth of well-defined crystal morphologies under mild reaction conditions, which is crucial for optimizing electrochemical performance. Meanwhile, the solid-state reaction technique, though more conventional, is recognized for its scalability and cost-effectiveness, making it a practical choice for industrial applications. Together, these methods provide a comprehensive overview of versatile synthesis approaches for V_2_O_5_.

#### 5.1.1. Sol–gel technique (method)

**Fig. 14:**
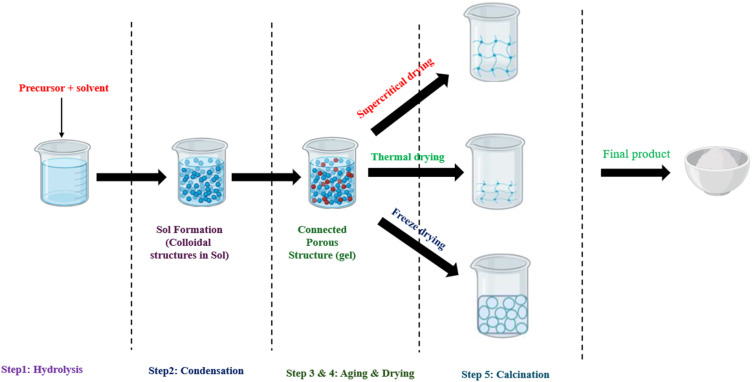
Process diagram and overview of the sol–gel technique for material synthesis.

The sol–gel technique is a versatile method that allows precise control over the morphology and particle size of materials at the molecular level. Although various synthesis methods can produce large volumes of nanomaterials, the sol–gel technique is widely employed at the industrial level due to its numerous advantages.^118,119^ These include its ability to produce high-quality nanoparticles with uniform size, as well as the capability to synthesize multiple types of nanoparticles simultaneously.^118,120,121^ This simultaneous production is particularly valuable for synthesizing multiple metal precursors by allowing different compositions in a single run at an industrial scale. Additionally, the sol–gel method yields homogeneous compositions materials with exceptionally high purity, often exceeding 99.99%.^118,122–124^ The sol–gel technique is also energy-efficient, operating at relatively low temperatures ranging from 70 to 320 °C, which contributes to its cost-effectiveness.^118,125,126^ The process employed in the sol–gel technique is classified as a bottom-up synthesis method, where a series of irreversible chemical reactions occur to produce a desired product.^127,128^ During this process, the homogeneous precursor material, or “Sol”, undergoes a transition to form a gel, characterized by a dense, three-dimensional network. This transformation into the gel is achieved through a compaction process, ultimately resulting in the formation of wet gel.^118,129,130^ The Fig. 14 below illustrates the various stages of sol–gel method. The use of sol–gel method for synthesizing V_2_O_5_ is particularly advantageous due to its ability to control homogeneity and purity at the molecular level.^131^ This method involves transitioning the system from a liquid “sol” (often colloidal suspension) to a solid phase known as a gel. For the synthesis of V_2_O_5_, sol–gel process occurs through the hydrolysis and polymerization of metal alkoxides, resulting in a gel-like network. During hydrolysis, the precursor, such as V_2_O_5_, is dissolved in a chosen solvent and then condensed to form a sol, a colloidal suspension of nanoparticles.^132,133^ The gradual transition to a gel is achieved through polymerization and solvent loss, leading to a network of interconnected particles. The gel is then subjected to drying and calcination, producing the desired crystalline V_2_O_5_ structure.^133^ In the fabrication of V_2_O_5_ cathodes, the sol–gel method is highly recommended as it allows the incorporation of guest species into the V_2_O_5_ matrix, enhancing the cathode's electrochemical performance. Additionally, various studies have indicated that the production of nanostructured V_2_O_5_ with numerous active sites (high surface areas) significantly improves the capacity and the rate capability of the designed battery.^134–136^

Sol–gel method's process details:^136^

• Dissolution of vanadium precursors such as V_2_O_5_ or vanadium oxytriisopropoxide in a solvent like alcohol or water.

• Phase transition occurs in two steps: hydrolysis followed by condensation to form a gel.

• Formation of crystalline V_2_O_5_ structure after the gel is stabilized (aged), followed by drying and then thermal treatment (calcination).

• Control of morphology, including composition, homogeneity, and particle size of the final product, to ensure the material is suitable for battery application, where uniformity is critical parameter.

*Advantages*:^134^

• Production of homogeneous mixing at the molecular level.

• Production of fine, uniform particles with excellent purity.

• Lower processing temperatures compared to other methods.

*Disadvantages*:^134^

• Time-consuming due to the aging and drying steps.

• Potential shrinkage and cracking during the drinking process.

The process diagram in Fig. 14 outlines the sequential steps involved in implementing a complete sol–gel technique for material synthesis.

#### 5.1.2. Hydrothermal technique

Hydrothermal synthesis is a technique utilized under high temperatures and pressures to process nanoparticles. This method has been extensively explored due to its advantages in producing nanostructured materials for various applications, including electronics, optoelectronics, catalysis, ceramics, magnetic data storage, biomedicine, and biophotonics. The hydrothermal approach is particularly effective for synthesizing nanodispersed and highly homogeneous nanoparticles. It also applicable in the production of nano-hybrid and nanocomposite materials.^137,138^ The hydrothermal (or solvothermal) processing technique is characterized as a heterogeneous reaction occurring in the presence of aqueous solvents or mineralizers under high pressure and temperature conditions. This method enables the dissolution and recrystallization of materials that are relatively insoluble under standard conditions, such as room temperature and atmospheric pressure.^137^

**Fig. 15:**
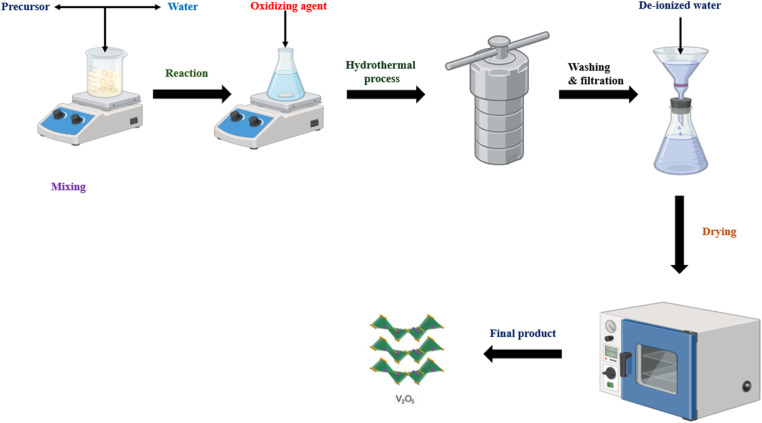
Process diagram and step-by-step overview of the hydrothermal synthesis technique for V_2_O_5._

This review examines the hydrothermal (solvothermal) processing technique, particularly in the context of synthesizing V_2_O_5_ materials. Fig. 15 presents a process diagram accompanied by a step-by-step overview of the hydrothermal synthesis technique.

##### 5.1.2.1. Hydrothermal synthesis of vanadium pentoxide (V_2_O_5_)

V_2_O_5_, a metal oxide, has been explored for various applications, including catalysis, energy storage, and electronics. V_2_O_5_ is synthesized through various methods, including hydrothermal synthesis. In this technique, vanadium precursors, such as vanadium oxides or vanadium salts, are dissolved in aqueous solution under controlled temperature and pressure conditions. Hydrothermal synthesis is advantageous as it allows for precise control of reaction environment, resulting in nanostructured V_2_O_5_ with unique morphology and phase purity.^139,140^ A typical hydrothermal synthesis involves preparing an aqueous solution of a vanadium precursor, commonly ammonium metavanadate (NH_4_VO_3_) or vanadium oxytrichloride (VCl_3_), along with a small amount of surfactant, such as cetyltrimethylammonium bromide(CTAB, C_19_H_42_BrN), or a pH-modifying agent like hydrochloric acid (HCl). These additives play a critical role in modulating nucleation kinetics and stabilizing specific crystal facets, thereby directing the growth of well-defined nanostructures. Depending on the reaction conditions, this method enables the formation of ultrathin nanosheets (typically 150–200 nm across), high-aspect ratio nanorods (up to 1 μm), or even complex flower-like assemblies. Such morphologies are strategically engineered to optimize ion transport and maximize surface to volume ratio, features that are essential for enhancing electrochemical performance in battery and catalytic systems, as demonstrated by Lui *et al.*^141^ and Liang Qiao.^142^ After homogenization, the solution is transferred into a teflon-lined stainless steel autoclave and heated at 180–220 °C for 12–24 hours under autogenous pressure, typically ranging from 1 to 2 MPa. Under these near-supercritical aqueous conditions, vanadium pentoxide completely dissolves and recrystallizes into an orthorhombic V_2_O_5_ phase. Various studies systematically investigated the influence of temperature on the resulting product morphology. At lower temperatures (∼150–180 °C), the hydrothermal process predominantly yields two-dimensional nanosheets, while higher temperatures (∼200–220 °C) favour the formation of one-dimensional nanorods.^143–145^ After synthesis, the product is allowed to cool naturally to ambient temperature. The resulting suspension is then vacuum-filtered, and the collected solids are thoroughly washed with deionized water to remove any residual salts or surfactants. The material is gently dried at approximately 60 °C to preserve the integrity of the nanostructure, followed by annealing at 400–500 °C for 2 to 4 hours in air. This thermal treatment eliminates amorphous impurities, sharpens X-ray diffraction (XRD) peaks, and promotes the formation of a well-ordered orthorhombic V_2_O_5_ lattice, an essential criterion for achieving reproducible and high-rate electrochemical performance in AZIBs.^146,147^ From an economic perspective, the hydrothermal production of V_2_O_5_ can be cost-competitive, provided that key process parameters are effectively optimized. A major contributor to the overall operating cost is the price of vanadium salts, which typically accounts for 50–60% of total expenses. This cost can be significantly reduced by sourcing precursors such as NH_4_VO_3_ or VCl_3_ for metallurgical by-products, namely spent petroleum catalysts or steel slag, where vanadium recovery is more economical, estimated at approximately $2–3 per kg of V_2_O_5_ equivalent. Furthermore, implementing closed-loop wastewater treatment systems, such as mild precipitation or ion-exchange columns, can recover 70 to 80% of dissolved vanadium. This not only reduces raw material consumption but also mitigates exposure to market price fluctuations.^148,149^ Another critical parameter in process optimization of hydrothermal synthesis is the net energy consumption. Maintaining hydrothermal conditions, typically 180–220 °C and 1–2 MPa, does not exceed 30% of the total operating costs in a pilot-scale system producing approximately 100 kg per day of material. However, integrating heat-recovery strategies, such as utilizing waste heat from on-site furnaces, can reduce energy consumption by estimated 15–20%. Such as measures not only improve overall efficiency but also contribute to the economic and environmental sustainability of the hydrothermal synthesis process.^150,151^ Moreover, as reported by Liu *et al.*,^152^ the use of continuous-flow hydrothermal reactors (CFHRs) is essential for achieving space-time yields of approximately 1 kg of V_2_O_5_ per unit volume per day, nearly three times higher than those obtained with conventional batch autoclaves. This approach significantly increases production throughout while reducing capital expenditure, making it a promising route for scaling up hydrothermal V_2_O_5_ synthesis.^153^ In addition to the previously discussed parameters, the design of a mid-scale production system targeting an annual output of approximately 5 tonnes of V_2_O_5_ must also be considered. Such a system would require the integration of two additional Teflon-lined stainless-steel autoclaves, each with a capacity of 500 L, alongside downstream modules for filtration, washing, drying, and annealing. The estimated capital cost for acquiring these autoclaves fall within the range of USD 1.2–1.5 million, based on the proposed configuration. This scale-up strategy is critical for bridging laboratory-scale feasibility with industrial implementation.^154–156^ The economic viability of hydrothermal synthesized V_2_O_5_ hinges on the amortization of fixed capital costs over a defined production scale for instance 10 tonnes per annum. With efficient precursor recycling and integrated energy recovery systems, operating costs can be brought down to approximately USD 12–15 per kg, compared to the current market price of USD 25–30 per kg for battery-grade V_2_O_5_. At this rate, a monthly output of 300–500 kg is generally sufficient to reach the breakeven point. Sensitivity analyses suggest that a 20% reduction in precursor costs or a 10% improvement in energy efficiency could further lower the breakeven threshold to under 300 kg per month, enhancing the commercial appeal of the process. From an environmental perspective, hydrothermal synthesis of V_2_O_5_ is inherently cleaner than many alternative methods, as it relies exclusively on water as the reaction medium and generates virtually no volatile organic compounds (VOC) can be treated on-site using chemical precipitation, ion exchange, or membrane separation techniques, enabling recovery rates of up to 90%. This not only minimizes environmental impact but also avoids costly effluent disposal fees to circular resource utilization. The hydrothermal synthesis emerges as a technically robust and economically feasible route for producing high-purity, nanostructured V_2_O_5_ suitable for demanding applications such as rechargeable AZIBs, catalysis, and electronics. The method offers fine control over morphology and crystallinity, enabling the fabrication of tailored nanostructures that enhance electrochemical performance. Economically, process viability is achievable at moderate production scales, particularly when precursor recycling, heat integration, and continuous-flow operation are implemented. Cost analyses indicate breakeven thresholds below 300 kg per month under optimized conditions, with market prices allowing for healthy margins. Environmentally, the use of water as the sole solvent minimizes VOC emissions, while high rates of vanadium recovery from effluents supports closed-loop, low-waste manufacturing. Altogether, these technical and economic parameters affirm that hydrothermal synthesis is not only scalable but also aligned with sustainable goals, making it a promising platform for the commercial production of battery-grade V_2_O_5_.

#### 5.1.3. Solid-state reaction

**Fig. 16:**
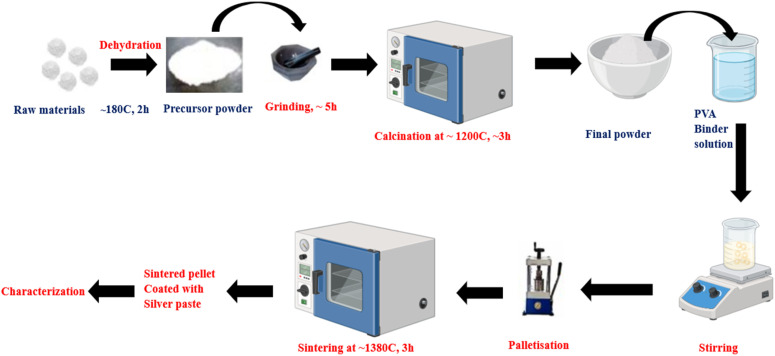
Diagram of solid-state reaction process, illustrating the sequence of steps involved in the synthesis, including the mixing, grinding, and high-temperature treatment of solid reactants to form the desired product. The diagram highlights key stages such as diffusion, particle bonding and the formation of a homogeneous phase.

The solid-state reaction is a widely used technique for synthesizing V_2_O_5_, involving the processing of solid precursors at elevated temperatures. This method is favoured for its simplicity, cost-effectiveness, and potential to produce high-purity materials.^157^Fig. 16 provides a detailed representation of the process description.

In a typical process, stoichiometric mixtures of vanadium precursors, commonly VO_2_, V_2_O_3_, vanadium oxalate (C_2_O_4_·2V_2_O_5_) or intermediate forms of V_2_O_5_, are subjected to rigorous mixing and high-energy milling to ensure a homogeneous, fine-grained powder. Such thorough comminution minimizes local concentration gradients and guarantees uniform diffusion of oxygen during subsequent thermal treatment, thereby promoting complete conversion to the orthorhombic V_2_O_5_.^158–160^ The calcination step is then carried out in an oxygen-rich atmosphere, with temperatures typically ranging between 800 °C and 1000 °C. Under these conditions, lower oxidation-state vanadium species are progressively oxidized according to the reaction.
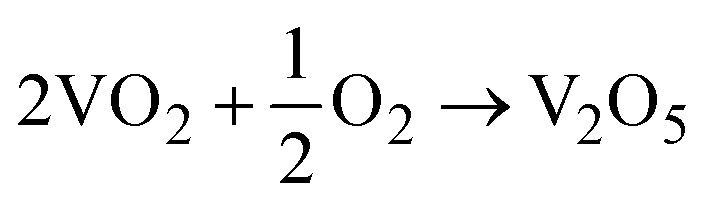


Precise control over heating rates (*e.g.*, 2–5 °C min), dwell times (4–12 h), and peak temperature is essential: slower ramping enhances crystal domain size and phase purity, while faster heating may induce nanocrystalline V_2_O_5_ domains that can shorten Zn^2+^ diffusion paths in battery in battery applications.^161–163^ After calcination, the material is cooled at a controlled rate to prevent thermal stresses that could fracture grains or introduce residual strain. A secondary milling step, often *via* ball or jet milling, is then employed to reduce agglomerated particles to a submicron-scale or micrometer-scale powder, typically 1–5 μm, which affords high surface area and ideal slurry and ideal slurry rheology for electrode fabrication.^161^ From a commercial perspective, solid-state synthesis capitalizes on readily available equipment, rotary kilns or continuous-belt furnaces, and establishes process controls, enabling throughput on the order of hundreds of kilograms per hour. Because the reaction depends only on thermal energy and simple precursor powders, capital expenditure remains moderate, and operating costs are dominated by energy consumption for heating. Recent techno-economic analyses estimate production costs for battery-grade V_2_O_5_ (≥99.9 wt%) to be approximately 5 to 8 USD per kg, contingent upon local energy costs and scale of operation.^164,165^ Crucially, the synthesis process yields materials with impurity levels below 0.1 wt%, a threshold essential for AZIB cathodes. Even trace metal contaminants can catalyse undesirable side reactions, including the hydrogen evolution reaction (HER) and dissolution of vanadium-based active materials, ultimately leading to accelerated capacity degradation and poor cycling stability.^11,113,166^ However, several challenges must be addressed so that solid-state V_2_O_5_ can satisfy all the stringent performance requirements of the next generation zinc-ion batteries. First, controlling particle morphology and crystallite size on an industrial scale remains beyond trivial. Conventionally produced V_2_O_5_ often features plate-like particles several micrometers thick, also this limits Zn^2+^ insertion kinetics. In order to overcome this, hybrid approaches, for example if they integrate spray pyrolysis coupled with solid-state calcination, have been developed so they can generate near-spherical particles, usually on the order of ∼1–2 μm, characterized through narrow size distributions, which thereby improves electrode packing density along with ionic diffusion pathways.^167,168^ Second, introducing also a controlled concentration of oxygen vacancies (V_2_O_5−*x*_), which can markedly improve electronic conductivity and rate capability, requires some careful modulation of O_2_ partial pressure or else addition of sacrificial carbon during calcination. Even though they do well show such defect engineering at the laboratory scale, translating such to continuous furnaces with high volume has yet to be fully realized.^169^ A high thermal budget (800–1000 °C for several hours) greatly contributes to energy consumption and CO_2_ emissions, third. Mechanochemical activation, where intense pre-milling induces partial amorphization, can reduce the required calcination temperature down to 300–500 °C, and it cuts energy use by up to 40% while still yielding phase-pure V_2_O_5_.^170^ Finally, V_2_O_5_ is generally reclaimed from spent cathode material through acid leaching or thermal reoxidation. Therefore, establishing closed-loop recycling protocols will be critical to minimize environmental impact also secure material supply.^9,171^

### 5.2. Characterization techniques

Following the synthesis of V_2_O_5_, the material undergoes various characterization techniques, each serving a specific purpose. For instance, X-ray diffraction (XRD) is used to confirm the purity of the synthesized V_2_O_5_ cathode material; scanning and transmission electron microscopy (SEM & TEM) are employed to determine particle morphology; and specific surface area analysis is conducted using the Brunauer–Emmett–Teller (BET) method to assess the surface properties.^157^ Despite the synthesis methods reviewed and the characterization of V_2_O_5_ as a cathode material for AZIBSs, several challenges persist. These include volume expansion after repeated intercalation/deintercalation of Zn^2+^ cations due to limited interlayer spacing, leading to cathode deformation, and dissolution of V_2_O_5_ in the electrolyte which forms by-products that contribute to capacity fading. To enhance the electrochemical performance of the cathode and ultimately produce high-performance batteries, several modifications strategies have been proposed.^73^ The following section will discuss the various strategies currently being employed.

### 5.3. Modification strategies

The cathode plays a pivotal role in the design of high-performance batteries, making the enhancement of its electrochemical properties essential for achieving superior battery performance. This section provides an in-depth analysis of the modification strategies used to optimize the electrochemical performance of vanadium pentoxide (V_2_O_5_) cathodes in aqueous zinc-ion batteries (AZIBs). Various techniques are currently employed to improve the structural integrity, electrical conductivity, and ionic diffusion of V_2_O_5_ within the cathode material, each of which is critical for the development of high-performance batteries.

This section aims to systematically present these strategies, which are integral to the fabrication of advanced cathodes and, ultimately, high-efficiency batteries.

#### 5.3.1. Guest species intercalation

**Fig. 17:**
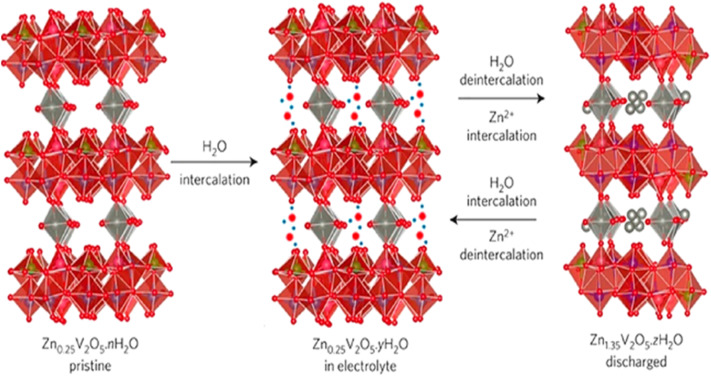
Co-intercalation of Zn^2+^ cations and water molecules into the V_2_O_5_ structure, illustrating the simultaneous insertion of both ions and water molecules.^79^

Guest species are incorporated into V_2_O_5_*via* pre-intercalation techniques to stabilize its structure and increase interlayer spacing, thereby enhancing its electrochemical performance. Various species are pre-intercalated into the V_2_O_5_ matrix before cathode synthesis, including metal ions from alkali, alkaline earth, and transition metals; organic molecules such as carbon black, carbon nanotubes (CNTs), reduced graphene oxide(rGO), and carbon cloth; as well as polymers like polyaniline (PANI), polyethylene oxide (PEO), and poly(3,4-ethylenedioxythiophene) (PEDOT). Additionally, there is ongoing research into the incorporation of quinone-based polymers and sulfonic acid groups into V_2_O_5_ to develop composite cathodes for AZIBs.^18,33,172,173^ The pre-intercalation of metal ions and water molecules effectively enlarges the interlayer spacing, facilitating enhanced ion transfer kinetics during charge–discharge cycles. Meanwhile, the incorporation of polymers improves the flexibility and cycling stability of the V_2_O_5_ cathode, contributing to its overall performance in AZIBs.^18,33^ To enhance the electrochemical performance of V_2_O_5_, a study investigated the co-intercalation of Zn^2+^ cations and water molecules (H_2_O) into the layered V_2_O_5_ structure during the discharge process. The co-intercalation leads to the formation of a reversible phase transition from V_2_O_5_ to a novel layered phase, denoted as Zn_*x*_V_2_O_5_·*n*H_2_O. During the charging process, this new phase reverts to V_2_O_5_, resulting in a unique morphological transformation. After 100 cycles, the material evolves into nanosheets with a porous structure. This porous structure significantly increases the specific surface area from 13.6 to 118.4 m^2^ per gram, thereby providing more active sites for ion exchange. The enhanced specific surface area improves Zn^2+^ ion transfer kinetic, ultimately boosting the electrochemical performance of the V_2_O_5_ cathode.^18,72^ Zhang *et al.* synthesized a V_2_O_5_ cathode that demonstrated an impressive capacity of 470 mA h per gram at current density of 0.2 A per gram, along with 91.1% capacity retention after 4000 cycles at a current density of 5 A per gram. The battery cell utilized a 3 M concentration of Zn(CF_3_SO_3_)_2_ as the electrolyte. The remarkable electrochemical performance is attributed to the co-intercalation of Zn^2+^ cations and H_2_O molecules, as well as the specific concentration of Zn(CF_3_SO_3_)_2_, which raises the oxygen evolution potential to 2.5 V. This increase helps prevent side reactions typically associated with water molecules within the operating voltage plateau of 0.2 to 1.6 V during the charging process. Additionally, the presence of H_2_O molecules in the V_2_O_5_ layered structure facilitates the easy diffusion of Zn^2+^ cations, thanks to the charge-shielding effect provided by the water molecules.^72^ The Fig. 17 outlines the significance of the co-intercalation of Zn^2+^ cations and water molecules into the V_2_O_5_.

**Fig. 18:**
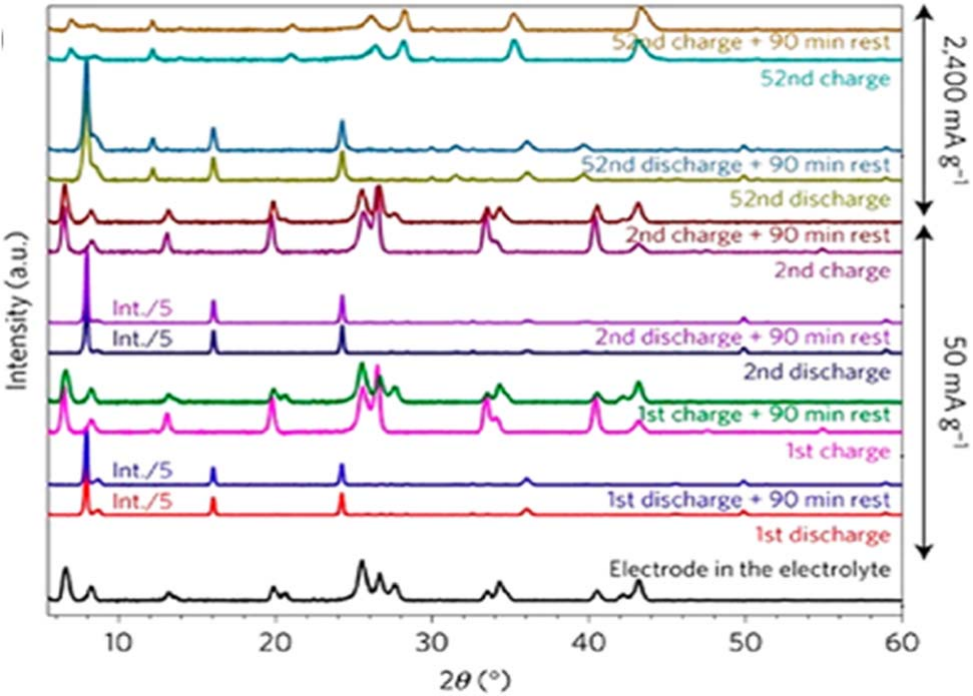
XRD patterns of Zn_0.25_V_2_O_5_·*n*H_2_O at various stages of electrochemical cycling.^79^

Despite the co-intercalation of Zn^2+^ cations and H_2_O molecules, the use of V_2_O_5_ has been associated with low electrical conductivity and poor cycling stability. These issues arise due to the structural instability of V_2_O_5_ and the dissolution of the cathode material into the electrolyte, leading to significant capacity fading after extended cycling periods.^18,174^ The Fig. 18 illustrates the characterization of the co-intercalation of Zn^2+^ and water molecules into V_2_O_5_ using the XRD technique at various stages of electrochemical cycling.

**Fig. 19:**
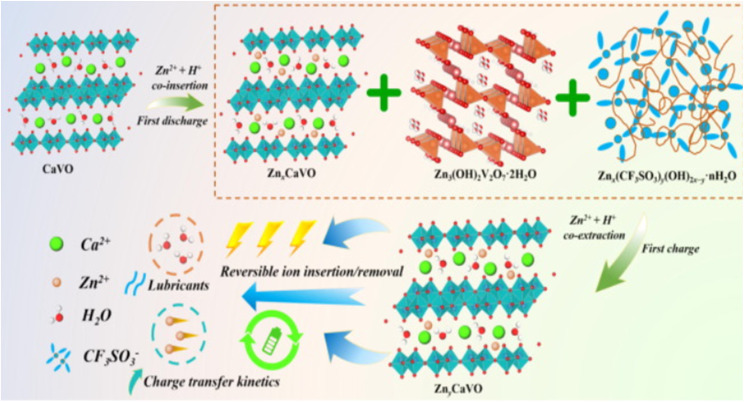
Diagram illustrating the pre-intercalation of Ca^2+^ ions into the V_2_O_5_ structure, showing how Ca^2+^ ions expand the interlayer spacing and enhance ion transport pathways.^176^

In addition to the promising intercalation of water molecules, another class of materials that has demonstrated significant advantages includes metal cations such as sodium (Na^+^), potassium (K^+^), and calcium (Ca^2+^). These cations contribute to the expansion of the interlayer spacing and enhance the ability of the V_2_O_5_ crystal structure due to their larger ionic size, which also minimizes the electrostatic interactions between the intercalated Zn^2+^ cations and the V_2_O_5_ structure, leading to an increase in interlayer spacing by 3.5 Å when used as a cathode with an aqueous electrolyte. This expanded interlayer spacing significantly improves the ion transfer kinetics of Zn^2+^ during charge–discharge cycles. Moreover, the electrical conductivity of the Ca^2+^ pre-intercalated V_2_O_5_ cathode is reported to be up to four times higher than of non-intercalated V_2_O_5_ cathode. As a result of this pre-intercalation, the battery exhibited a capacity of 340 mA h per gram at a current density of 0.2 A per gram, along with 96% capacity retention over 3000 cycles at a high current density.^18,79,175^ The pre-intercalation of Ca^2+^ is not without its challenges, particularly regarding capacity fading when the battery is discharged to 0.6 V, as reported by Xia *et al.* At this voltage, a peak split is observed, indicating significant degradation of the crystal structure of the cathode. This degradation is primarily due to the excessive insertion of Zn^2+^ cations into the interlayer spacing of the Ca^2+^ pre-intercalated V_2_O_5_, leading to substantial stress accumulation within the material. At this voltage, the crystal structure of the cathode accommodates nearly 1.5 Zn^2+^ (ref. 1 and 31) cations per V_2_O_5_ molecule, which, despite not causing massive structural distortion, still results in considerable stress and subsequent capacity fading.^175^Fig. 19 demonstrates how the intercalation of Ca^2+^ cations into V_2_O_5_ enhances the interlayer spacing, thereby improving the electrochemical performance of V_2_O_5._

**Fig. 20:**
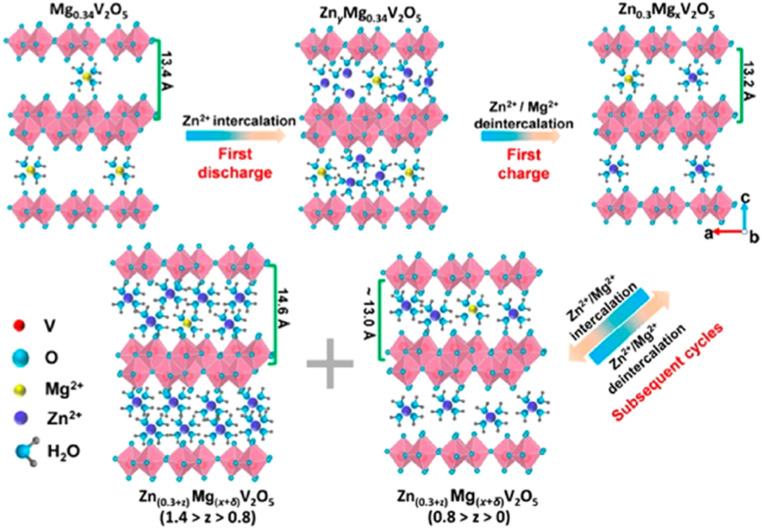
Schematic illustration of the pre-intercalation of Mg^2+^ ions into V_2_O_5_ structure, demonstrating how Mg^2+^ ions increase interlayer spacing and improve ion diffusion channels.^177^

The pre-intercalation of magnesium to form Mg_0.34_V_2_O_5_·*n*H_2_O is particularly intriguing due to its occurrence *via* displacement intercalation reaction mechanism. During the discharge process, the insertion of Zn^2+^ cations leads to the replacement of most Mg^2+^ cations, resulting in the formation of Zn_0.3_Mg_*x*_V_2_O_5_. Upon charging, the displaced Mg^2+^ cations are reinserted into the Zn_0.3_Mg_*x*_V_2_O_5_ structure.^177^ This displacement intercalation reaction was first observed during the pre-intercalation of silver into V_2_O_5_ (forming Ag_0.4_V_2_O_5_ cathode) structure to accommodate Zn^2+^ cations.^178^ The pre-intercalation of Mg^2+^ cations in V_2_O_5_ significantly increases the interlayer spacing to approximatively 13.4 Å and results in a wide working voltage plateau, ranging from 0.1 to1.8 V *versus* Zn^2+/^Zn, as reported by Ming *et al.*^177^ The Fig. 20 is a prime example of how the pre-intercalation of Mg^2+^ and the co-intercalation of Zn^2+^ and Mg^2+^ increase the interlayer spacing of the V_2_O_5_ cathode, resulting in enhanced electrochemical performance.

In addition to the types of pre-intercalations, the incorporation of transition metals represents another effective strategy to enhance the electrochemical performance of V_2_O_5_ cathodes. Transition metals such as cobalt (Co), copper (Cu), iron (Fe), manganese (Mn), and nickel (Ni) have been extensively pre-intercalated to expand the interlayer spacing, improve ionic transport, increase electronic conductivity, enhance cyclability, and reduce polarization. This process also stabilizes the cathode structure by forming stronger chemical bonds between the pre-intercalated transition metals and the [VO_*n*_] layers, which are more robust than the weak van der Waals interactions, thereby ensuring higher structural stability during cycling and reducing voltage degradation during self-discharge.^179,180^ Additionally, the significant presence of V^4+^ ions, particularly with the introduction of Mn^2+^, further catalyses electrochemical reactions. The larger ionic radius of the V^4+^ cation enlarges the interlayer spacing, while its 3d orbital electrons contribute to improved electrical conductivity. Similar effects have been observed when transition metals such as nickel and cobalt are pre-intercalated into hydrated V_2_O_5_.^180^ Studies have shown that transition metals exhibit a catalytic effect on electrochemical redox reactions, and their-intercalation can also lead to transformations in the crystal structure of the cathode material.^70,181^ For instance, Oka *et al.*, developed a cathode material in which zinc, a transition metal was pre-intercalated into a V_2_O_5_ matrix. This cathode was characterized by two-dimensional V_2_O_5_ bilayers with Zn^2+^ ions and water molecules intercalated between the layers. The modification strategy led to a reversible capacity of approximately 282 mA h per gram at a current density of 300 mA per gram and an impressive capacity retention rate of 81% after 1000 cycles at a current density of 2400 mA per gram in a 1 M ZnSO_4_ electrolyte solution. These exceptional electrochemical performances are attributed to two primary factors: the formation of ZnO_6_ and the presence of intercalated water molecules, which contribute to the stability of the layered structure and facilitate the interaction of additional Zn^2+^ ions. Additionally, the resulting nanobelt morphology enhances ion transfer kinetics and provides efficient interfaces between the electrode and the electrolyte, improving both ionic and electronic transport. Structural stress experienced during repeated Zn^2+^ insertion and extraction also contributed to the nanostructured framework, thereby enhancing cycling stability (Table 2).^18,182^

**Table 2:** Comparison of the size and effects of the alkaline earth metal cations and pre-intercalated ions in the V_2_O_5_ structure, highlighting how different ion sizes influence interlayer spacing, structural stability, and electrochemical performance

Ion	Ionic radius^183^ (Å)	Hydrated radius^184^ (Å)	*x* in M_*x*_V_2_O_5_·*n*H_2_O	Interlayer spacing (Å)	Capacity retention|cycles|current density	*D*_Zn^2+^_ (cm^2^ s^−1^)	Reference
Lithium	0.69	3.82		13.8	232 mAh g^−1^|500|5 A	10–9 to 10–8	185
Sodium	1.02	3.58	0.29	11	83%|1800|10 A g^−1^	10–9 to 10–8	61
Potassium	1.38	3.31	0.17	9.9	92%|3000|5 A g^−1^	10–13	186
Magnesium	0.72	4.4	0.34	13.4	97%|2000|5 A g^−1^	10–9 to 10–8	177
Zinc	0.75	4.3	0.25	10.2	81%|1000|2.4 A g^−1^	10–10 to 10–9	79
Calcium	1.00	4.12	0.25	10.6	96%|3000|80 C	10–9 to 10–8	175
Aluminium	0.53	4.75	0.84	13.4	100%|3000|4 A g^−1^	10–11	187

**Fig. 21:**
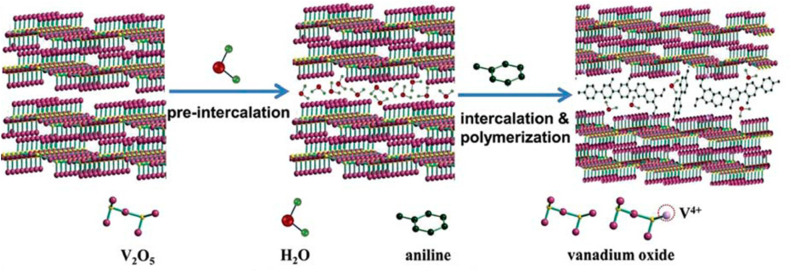
Schematic diagram of the polyaniline pre-intercalated V_2_O_5_ composite synthesis.^190,191^

**Fig. 22:**
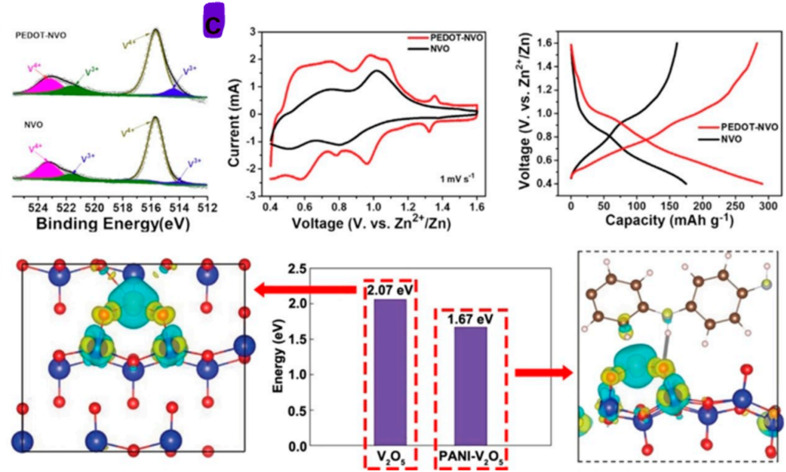
The electrochemical performance evaluation of polyaniline (PANI) pre-intercalated V_2_O_5_ as a cathode material.^191^

Lastly, but equally important, is the modification of both the zinc anode and the cathode materials using polymer-based materials. Polymers have recently gained significant attention as vital materials for modifying cathodes in the design of AZIBSs, particularly the V_2_O_5_ cathode. When pre-intercalated into the V_2_O_5_ matrix, polymers enhance the electrochemical performance and improve the structural integrity and stability of the cathode. These improvements are attributed to the inherent advantages of polymers, such as high energy density, favourable electrochemical properties, mechanical stability, excellent electrical conductivity, and inherent safety.^175^ Following polymer pre-intercalation, the V_2_O_5_ cathode exhibits enhanced conductivity, increased interlayer spacing, stabilization of active materials, and an abundance of active sites. In addition to enhancing the electrochemical performance of the cathode, polymers also serve as a protective layer for the zinc anode, preventing electrolyte-induced erosion at the interface between the aqueous electrolyte and the zinc anode. This protective layer facilitates ion transportation, improving ion transfer kinetics, and helps prevent volume expansion in both electrodes.^188^ The use of polymers in this modification strategy is particularly valuable due to their intrinsic properties, such as electrochemical inertness, ease of large-scale production, high resistance to deformation, and the ability to incorporate designable functional groups.^175,188^ Moreover, the flexibility structure of polymers compensates for volume expansion during the repeated insertion and extraction of Zn^2+^ cations from the cathode during the battery operation. Polymers also play a crucial role in accommodating inevitable side reactions, mitigating the dissolution of electrodes, and preventing electrode damage. Additionally, they provide a source of active materials, leading to an increase in active sites that further promote ion transfer kinetics.^67,189^ Currently, three types of polymers are employed as cathode modifiers: conductive polymers, redox, and carbon composite polymers.^175^ The Fig. 21 illustrates the incorporation of polyaniline into the V_2_O_5_ cathode to improve its interlayer spacing, which ultimately enhances the electrochemical performance, as shown in Fig. 22.

#### 5.3.2. Nanostructuring engineering

Nanostructure modification strategy is a technique used to manufacture V_2_O_5_ with structures such as nanowires, nanobelts, or nanosheets, which significantly enhance the electrochemical performance of battery electrodes.^192–195^ This enhancement is primarily due to the increased surface area and shorter ion diffusion paths, leading to improved rate capability and cycling stability. Various studies have demonstrated that nanostructuring is a critical strategy in the production of high-performance electrode materials for batteries.^195,196^ The resulting electrodes benefit from a high specific surface area, which increases the contact between the V_2_O_5_ cathode and the electrolyte at the interface, thereby reducing electrochemical polarization. Additionally, this approach endows the cathode with abundant active sites and further enhances its rate capability.^192,197^ The investigation by Yao *et al.* demonstrates that nanostructuring engineering produces various types of V_2_O_5_ cathode materials, organized within nano-confinement dimensions. These nanostructured V_2_O_5_ cathodes are classified into different categories based on their dimensional arrangement: zero-dimensional (0D), one-dimensional (1D), two-dimensional (2D), and three-dimensional (3D) nanomaterials.^195^

Zero-dimensional (0D) nanostructuring of V_2_O_5_ is one of the most widely employed synthesis methods for designing V_2_O_5_ cathodes, as this approach significantly enhances ion transfer kinetics by shortening the diffusion path of cations such as Zn^2+^ and Li^+^ during battery operation. This has been particularly observed in lithium-ion batteries (LIBs) utilizing nanostructured V_2_O_5_ cathodes. Moreover, this nanostructuring technique effectively minimizes the impact of concentration polarization in the solid state, especially during charge–discharge cycles, which contributes to achieving a high specific charge capacity within the same voltage plateau.^195,198^ Additionally, the nanostructuring of the cathode provides a large contact surface area at the electrode–electrolyte interface, leading to higher electrochemical activity. As a result, V_2_O_5_ nanostructured cathodes exhibit enhanced cycling stability and superior rate capabilities compared to commercially available microstructured V_2_O_5_.^195,199^

One-dimensional (1D) nanostructuring is a technique used to modify electrode nanostructures, resulting in the formation of nanowires, nanorods, nanotubes, and nanobelts. These structures are characterized by a direct pathway along a microscale axis, which enhances ion transfer kinetics and improves the rate capabilities.^195,200,201^ Due to these advantageous properties, 1D nanostructuring has been extensively explored for the design of electrochemical energy devices.^202–204^ To modify the structure of V_2_O_5_ cathode material using 1D nanostructuring, several preparation methods are employed, including template growth, hydrothermal synthesis with post-heat treatment,^205–207^ chemical vapor transport, chemical vapor deposition (CVD), and heating of ball-milled V_2_O_5_ powders in air.^208,209^ For example, Patrissi *et al.* synthesized V_2_O_5_ nanorod arrays using a template growth method. In their experiment, precursors into the pores of microporous template membrane, which was later removed *via* chemical etching or thermal annealing.^205^ They used a polycarbonate filtration membrane (PC) as the template to fabricate polycrystalline V_2_O_5_ nanorod arrays. These nanorod arrays exhibited excellent capacity, significantly outperforming thin-film V_2_O_5_ electrodes at high rate of 200 °C, with capacity increasing up to four times at discharge rates between 500 °C and 1190 °C. Additionally, single-crystalline V_2_O_5_ nanorod arrays were fabricated using electrophoretic deposition combined with radiation track-etched hydrophilic PC membranes as templates. This resulted in V_2_O_5_ cathodes with lengths of approximately 10 μm and diameters ranging from 100 to 200 nm, exhibiting nearly unidirectional alignment over a large area. These single-crystalline V_2_O_5_ nanorods, which grew along the [010] direction, demonstrated an applicable current density approximately five times greater than that of sol–gel derived films. In lithium-ion batteries (LIBs), these cathodes could intercalate up to five times more Li^+^ cations compared to sol–gel derived films, highlighting their superior performance.^206,210,211^

Two-dimensional (2D) nanostructuring is a promising technique for enhancing electrochemical energy storage devices, particularly in the design and development of LIBs.^195,212^ This approach offers several advantages, including short diffusion paths Li^+^ cations and a large, exposed surface area that provides abundant active sites for cation insertion. This is particularly beneficial for the storage of larger cations, which require stability, plentiful active sites, and short diffusion paths for insertion and extraction without causing electrode deformation or volume expansion during battery operation.^195,213^ Li *et al.* investigated the synthesis of two-dimensional leaf-like V_2_O_5_ nanosheets using a straightforward sol–gel method combined with freeze-drying and annealing in air. The resulting V_2_O_5_ polycrystals comprised small nanorods and nanosheets with thicknesses of approximately 60–80 nm. This V_2_O_5_ cathode exhibited discharge capacities of 303, 273, 251, 219, and 160 mAh g^−1^ at current densities of 50, 200, 500, 1000, and 2000 mAh g^−1^, respectively. Demonstrating a decrease in discharge capacity with increasing current density. Furthermore, the developed cathode provided a high capacity of 104 mAh g^−1^ at an elevated current density of 5000 mA g^−1^. The modified V_2_O_5_ cathode facilitates rapid ion transfer due to its structure, which features short diffusion paths for efficient cation transport.^195,214^ While previous nanostructuring techniques like 1D and 2D modifications offer significant benefits, they also come with certain limitations, such as low dimensionality leading to high electrical resistance and reduced mechanical stability.^195^ Additionally, the nanometre-scale particle size presents challenges in achieving high packing density and complicates the design and processing of electrodes. These limitations highlight the advantages of employing three-dimensional (3D) nanostructuring techniques for V_2_O_5_ electrodes. The 3D nanostructured frameworks create quasi-continuous conductive networks that facilitate the efficient transport of both ions and electrons, significantly enhancing ion transfer kinetics and improving the structural stability of the electrode.^201,215–217^ The application of 3D nanostructure also mitigates particles agglomeration, leading to improved capacity. To modify V_2_O_5_ using 3D nanostructuring, various synthesis methods are employed, including solvothermal and hydrothermal techniques, which have been shown to yield structures such as porous microspheres, hollow microspheres, yolk-shelled and multi-shelled microspheres, micro flowers, as well as hollow micro flowers.^195,218^ For instance, Wang *et al.*^219^ utilized a 3D nanostructuring technique to produce porous, monodisperse V_2_O_5_ microspheres characterized by pores smaller than 30 nm and a high specific surface area of 31.2 m^2^ g^−1^. When used as cathode in lithium-ion batteries (LIBs), these nanostructured V_2_O_5_ microspheres exhibited enhanced electrochemical performance, high reversible capacity, and good cycling stability within voltage ranges of 2.05 V to 4.0 V and 2.5 V to 4.0 V (*vs.* Li^+^/Li).^195^ In another study, researchers synthesized V_2_O_5_ microspheres consisting of stacked platelets. The synthesis was performed at room temperature using V(OH)_2_NH_2_ as a precursor in an aqueous solution, followed by calcination. The resulting V_2_O_5_ microsphere cathode demonstrated a high discharge capacity, an excellent rate capacity of 223 mAh g^−1^ at current density of 2400 mA g^−1^, remarkable cycling stability, retaining 200 mAh g^−1^ after 100 cycles.^195,220^

#### 5.3.3. Surface coating

Surface coating is a widely employed modification technique to enhance electrical conductivity and prevent the dissolution of the cathode in aqueous electrolytes. This approach utilizes conductive materials such as carbon, graphene, and certain metal oxides. For instance, coating the surface of V_2_O_5_ with carbon, this process is known as carbon coating through which a conductive framework around the V_2_O_5_ particles is created. This carbon coating process is responsible for the reduction contact resistance, thereby significantly enhancing the overall electrical conductivity of the V_2_O_5_ cathode. Similarly, the use of graphene oxide (GO) as a coating material not only enhances the electrical conductivity but also reinforces the mechanical integrity and stability of the cathode structure.^221^

**Fig. 23:**
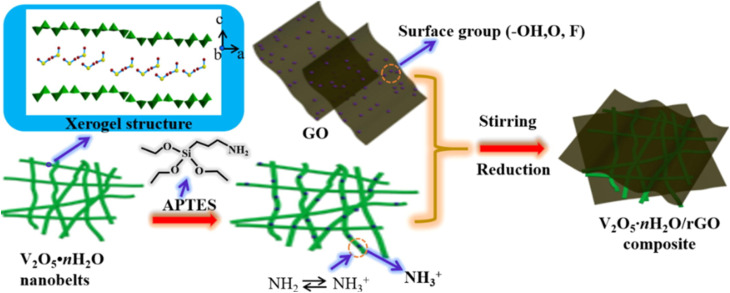
Schematic diagram, illustrating the surface coating modification of V_2_O_5_ using graphene oxide (GO).^226^

The surface coating of V_2_O_5_ has been extensively investigated as an effective strategy in the design of cathodes for LIBs to enhance their electrochemical performance. This approach has been particularly beneficial in improving the cycling stability and rate capability of the batteries.^222^ This technique offers a range of advantages for electrode materials, such as facilitating ion transfer at the surface of particles, especially when electron-conducting coatings are employed. Additionally, they help mitigate the aggregation of nanoscale active materials and buffer the mechanical stress within the electrode. It can also act as physical protective barriers, thereby enhancing the chemical stability of the electrode. Moreover, it can modify the surface chemistry of V_2_O_5_ cathodes, leading to improved overall performance. Importantly, these coatings prevent the dissolution of detached electrode particles under severe operating conditions, contributing to the long-term durability of the battery.^195^ Li and Zhou have recently reported that the application of surface coating techniques, when combined with nanotechnology, has been utilized to manufacture core–shell heterogeneous nanostructures. These advanced structures are designed to suppress aggregation, provide excellent electrical conductivity, and enhance ion transfer kinetics. Moreover, this approach significantly improves the rate capability of the battery, leading to superior overall performance.^223^ Odani *et al.* and Koltypin *et al.*,^224,225^ have investigated the surface coating of V_2_O_5_ nanoparticles with carbon by burning off carbon-coated V_2_O_5_ nanoparticles in air at 400 °C. The resulting carbon-coated V_2_O_5_ demonstrated exceptional capacity, superior rate capacity, and significantly enhanced stability during storage and cycling in alkyl carbonate solutions at elevated temperatures. These properties were markedly better compared to other types of V_2_O_5_ cathodes, such as bare V_2_O_5_ nanoparticles or V_2_O_5_ with micrometre-sized particles. The Fig. 23 illustrates the surface coating of V_2_O_5_ with graphene oxide (GO) as method for enhancing structural stability.

#### 5.3.4. Doping of V_2_O_5_

**Fig. 24:**
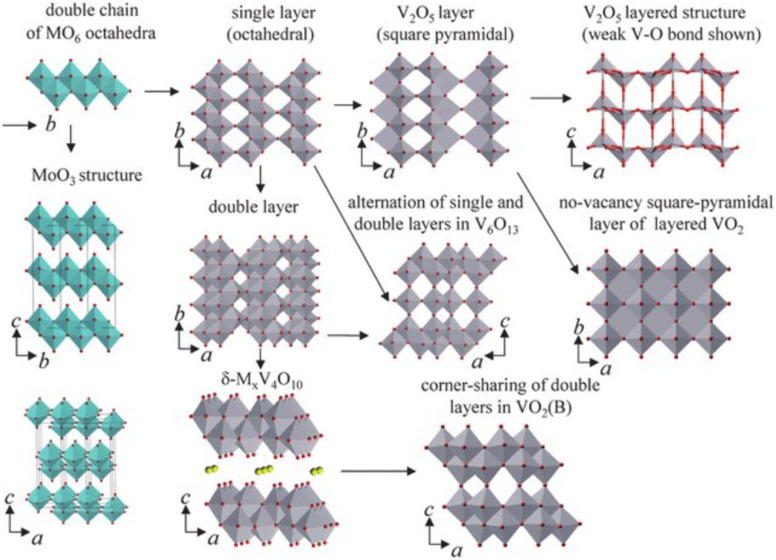
Different structural forms of molybdenum-doped vanadium pentoxide (V_2_O_5_).^235^

Doping is an effective modification technique widely used to significantly improve the electrochemical properties of metal oxides, polymers, and carbon-based materials when utilized as host materials in energy storage devices.^80^ This technique enhances the electrochemical performance of the host materials by incorporating elements such as manganese,^227^ nickel,^228,229^ copper,^230^ tantalum,^231^ or organic compounds. These dopants are extensively added to cation-host materials like V_2_O_5_, MnO_2_, and various polymers to boost their electronic conductivity. According to Saha *et al.* doping not only increases the electronic conductivity of the host materials but also enhances their pseudo-capacitive behaviour and structural stability, leading to improved overall electrochemical performance.^80,232^ The application of this technique results in netter rate capability and durability of the host cathode materials, making them more suitable for high performance energy storage applications.^80^ The doping modification strategy is highly effective for incorporating certain metals, such as molybdenum (Mo), due to its stable oxidation state (+6) and ionic radius of 0.73 Å, in comparison to vanadium(v), which has an oxidation state of +5 and an ionic radius of 0.68 Å. Various studies have demonstrated that doping V_2_O_5_ with Mo significantly enhances the structural integrity, optical properties, electrical conductivity, and overall electrochemical performance of the material. This improvement is attributed to Mo acting as donor-like defects within the V_2_O_5_ matrix. For instance, Prakash *et al.* investigated the doping of Mo into V_2_O_5_ thin films deposited on a nickel substrate *via* thermal evaporation. The process was carried out at a substrate temperature of 250 °C, with Mo concentrations of 2%, 4%, and 6%, and a current density of 1 mA cm^−2^. Their results revealed that V_2_O_5_ thin films doped with 4 at% Mo exhibited a maximum specific capacitance of 175 mF cm^−2^.^233^ Similarly, Shankar *et al.* utilized nickel (Ni) as a dopant for V_2_O_5_ synthesized through a sol–gel method, producing Ni-doped V_2_O_5_ nanorods. These nanorods achieved a specific capacitance of 152 F g^−1^, significantly higher than of pure V_2_O_5_. The enhanced electrochemical performance and stability of the doped V_2_O_5_ electrodes, as well as their improved electrical conductivity, are attributed to the presence of Ni-dopant cations, which facilitate better charge transport and structural resilience.^234^ The Fig. 24 outlines the modification of V_2_O_5_ structure through the incorporation of molybdenum as a dopant.

#### 5.3.5. Defect engineering

Defect engineering is another effective modification strategy, wherein impurities are intentionally introduced into the cathode structure to alter its electronic configuration, thereby impacting the electrochemical reactions that occur within the electronic structure of the electrode.^33,236^ This technique is employed to improve the electrochemical performance of cathode materials. By modifying the electronic structure, defect engineering enhances the conductivity and creates abundant active sites that facilitate the insertion and extraction of Zn^2+^ ions into and from the V_2_O_5_ cathode. As a result, V_2_O_5_ cathodes subjected to defect engineering exhibit an altered local electronic structure of transition metal elements, leading to increased conductivity and improved ion transport.^33,237^ Various studies have shown that defect engineering affects the Gibbs free energy of Zn^2+^ ions adsorbed onto the surface of defect-engineered V_2_O_5_ cathodes, resulting in more efficient Zn^2+^ ion insertion/extraction.^33,237,238^ For instance, Du *et al.* reported that this modification strategy not only enhances ion mobility but also stabilizes the cathode during cycling, thereby contributing to higher electrochemical stability.^239^ Defect engineering is typically implemented through a technique known as point defects, which can be applied at different sites using either anions or cations, when anions are used to modify the electronic configuration of the cathode, the technique is referred to as “anionic defects,” while the use of cations results in “cationic defects.” Both approaches offer unique advantages by selectively tuning the electronic structure, improving the cathode's overall performance in zinc-ion batteries.^33^

Anionic defect engineering is a modification strategy that involves introducing defects using anions. One commonly employed method within this strategy focuses on the use of oxygen, often referred to as oxygen defect engineering. Oxygen defects play a crucial role in enhancing the conductivity of cathode materials by altering their electronic structure. This technique effectively reduces the interlayer stress and electronic repulsion, thereby lowering the migration and diffusion barriers for cations. As a result, ion transfer kinetics and charge transfer are significantly improved during the insertion and extraction of Zn^2+^ ions.^33,240^ In the design of AZIBs, cathodes such as MnO_2_, V_2_O_5_, Prussian Blue, and polymers are oxygen-deficient environment, is typically sought to prevent side reactions like oxygen evolution reactions (OERs). However, the synthesis of oxygen-free cathodes presents challenges. While minimizing oxygen reduces unwanted side reactions, it also limits the formation of Zn–O bonds, leading to an excess of valence electrons migrating into the delocalized electron cloud, which improves conductivity. Nonetheless, oxygen-free (anoxic) cathodes are limited by the thermodynamic adsorption energy of Zn^2+^ in their surfaces. This limitation affects the utilization of electrochemically active sites, resulting in suboptimal performance.^33,241^ Unmodified cathodes, as reported by Zhao *et al.* exhibit a lower thermodynamic state due to the insufficient Gibbs free energy required for the intercalation/deintercalation of Zn^2+^. This insufficient energy reduces the capacity of host cathode material to accumulate Zn^2+^ ions, while also depleting active sites through the formation of Zn–O bonds, leading to capacity fading. This highlights the critical importance of anionic defect engineering, particularly through oxygen defect engineering. By deliberately introducing oxygen vacancies, the Gibbs free energy associated with Zn^2+^ adsorption near these vacancies can be adjusted to a thermodynamically neutral value, enhancing the electrochemical performance of the cathode.^242^ An investigation into oxygen defect engineering using σ-MnO_2_ cathodes revealed that the Gibbs free energy of Zn^2+^ adsorption was reduced to a thermal neutral value of approximately 0.05 eV. These decreases facilitated abundant active sites for ion transport and enabled faster Zn^2+^ insertion/extraction. Moreover, the diffusion pathway in the oxygen-deficient σ-MnO_2_ was notably shorter than in its anoxic counterpart, contributing to enhanced electrochemical performance.^243^ According to the study conducted by Zhang *et al.*, the incorporation of oxygen defect engineering into the fabrication of σ-MnO_2_ resulted in the transition of spinel ZnMn_2_O_4_ from a semiconductive to a conductive state by modifying the electronic structure near the Fermi level. This modification reduced the mobility barrier for Zn^2+^ cations around the oxygen defect sites, lowering it from 0.39 eV in the unmodified cathode to 0.24 eV. This reduction allowed Zn^2+^ cations to migrate more freely through the oxygen vacancies, leading to enhanced electrical conductivity and improved ion transfer kinetics.^33,244^ In another study, Liao *et al.* applied oxygen defect engineering to synthesize V_6_O_13_ through a self-assembly process based on solution redox, followed by heat treatment in an N_2_/H_2_ atmosphere (environment). The use of N_2_ was essential in creating an inert environment during synthesis. The modified V_6_O_13_ cathode exhibited significantly improved ion transfer kinetics, with faster Zn^2+^ diffusion rates and lower overpotential compared to its unmodified counterparts. The introduction of oxygen defects facilitated the insertion and extraction of Zn^2+^ ions, contributing to enhanced electrochemical performance.^33,245^ Additionally, it was observed that the unmodified V_6_O_13_ demonstrated uniform charge distribution, while the oxygen-defect-engineered cathode exhibited electron clustering near the oxygen vacancies in the lattice. This clustering was companied by a notable decrease in Gibbs free energy, from −2.736 eV to −0.38 eV, further supporting the role of oxygen defect engineering in improving the material's electrochemical properties.^33,246^

Cationic defect engineering is another important strategy that modifies the electronic properties of the cathode by incorporating specific cations. This approach weakens the electrostatic interaction between Zn^2+^ ions and the cathode, while also activating redox reactions within the cathode. The intentional inclusion of cations in the cathode structure enhances its electrochemical capacity by providing additional active sites for ion storage.^247^ Furthermore, the application of cationic defect engineering facilitates the insertion and extraction of Zn^2+^ ions and prevents volumetric expansion of the cathode, a common issue that leads to structural deformation and capacity fading due to the dissolution of active materials into the electrolyte.^33^ A study by Zhang *et al.* explored the synthesis of a ZnM_2_O_4_/C cathode, where manganese (Mn) was incorporated into using an ammonia-assisted oxidation precipitation method. The inclusion of Mn significantly reduced the electrostatic barrier for Zn^2+^ ion diffusion within the spinel framework. This modification resulted in improved Zn^2+^ ion transfer kinetics and faster electrode dynamics compared to the unmodified ZnM_2_O_4_, where Zn^2+^ ions experienced substantial electrostatic repulsion from Mn^2+^ ions at adjacent octahedral sites, creating a considerable diffusion barrier.^248^ In another investigation, Zhu *et al.* examined the effects of cationic defect engineering by introducing vanadium vacancies in a V_2_O_3_ cathode through a hydrothermal synthesis process. The modified V_2_O_3_ cathode exhibited a significantly lower Gibbs free energy of −1.34 eV, compared to 2.69 eV in the unmodified version. This reduction in Gibbs free energy facilitated enhanced insertion and extraction of Zn^2+^ ions, underscoring the benefit impact of vanadium vacancies on the electrochemical performance of the cathode.^249^

### 5.4. Position, distribution, and stability of dopant atoms in vanadium pentoxide (V_2_O_5_)

The defect engineering and elemental doping have emerged as effective strategies to enhance the structural integrity, electronic conductivity, and ion intercalation of V_2_O_5_ cathodes in multivalent ion batteries. Elemental doping can be achieved through two primary mechanisms including substitutional doping, where dopant atoms replace vanadium ions within the lattice, and interstitial doping, in which dopant atoms occupy the vacant interlayer spaces between the V–O layers. These modifications tailor the electronic structure and local environment of the host material, thereby improving its electrochemical performance and cycling stability.

#### 5.4.1. Substitutional doping

Substitutional doping of V_2_O_5_ is commonly achieved using dopant ion such as titanium(iv) (Ti^4+^), manganese(ii) (Mn^2+^), molybdenum(vi) (Mo^6+^), and chromium(iii) (Cr^3+^). These elements exhibit a strong tendency to occupy substitutional sites by replacing vanadium ions within the VO_5_ square pyramidal coordination environment. The selection of appropriate dopant is primarily guided by factors such as ionic radius, oxidation state, and coordination chemistry. To ensure efficient lattice integration and minimize the formation of strain or defect states, the ionic radius and valence of the dopant should closely match those of V^5+^. A close match reduces the energetic cost of substitution, facilitating seamless incorporation of the dopant into the host lattice. For instance, the substitution of V^5+^ (ionic radius ≈0.59 Å) with Ti^4+^ (ionic radius ≈0.605 Å) is thermodynamically favourable due to their comparable size, allowing for effective doping without significant lattice distortion.

#### 5.4.2. Interstitial doping

Alternatively, small-radius or low-valence cations such as lithium (Li^+^) and sodium (Na^+^) are often employed as interstitial dopants, particularly in hydrated or expanded phases of V_2_O_5_. These dopant ions occupy the interlayer voids between the V–O layers rather than substituting for lattice vanadium atoms. Their incorporation increases the interlayer spacing through modulation of the van der Waals interactions, thereby facilitating faster ion diffusion and improving the kinetics of Zn^2+^ intercalation. Furthermore, these interstitial dopants help to alleviate electrostatic repulsion between the inserted ions and the host lattice during charge–discharge cycles, contributing to enhanced structural stability and improved cycling performance of the cathode material. The stability of dopant atoms within the V_2_O_5_ host lattice depends on several interconnected factors including charge compensation, redox activity (redox behaviour), bonding enthalpy, lattice relaxation, and defect chemistry. In substitutional doping, vanadium atoms (typically V^5+^) are partially replaced by dopants species such as Ti^4+^ or Mn^2+^, which introduces local charge imbalances and triggers charge redistribution throughout the lattice. This often results in the partial reduction of adjoining V^5+^ to V^4+^, a process that enhances electronic conductivity through small-polaron hopping and stabilizes the local electronic environment. In parallel, bonding enthalpy plays a critical role in dopant stability; dopants that form strong metal–oxygen bonds comparable in strength to V–O bonds are thermodynamically more promising for lattice incorporation. Lattice relaxation and defect chemistry further influence dopant stability. First-principles calculations, such as density functional theory (DFT), have shown that dopant incorporation triggers local distortions that are defect-tolerant, thereby lowering the formation of energy of the doped structure. These local relaxations help stabilize metastable phases, including amorphous or xerogel-like V_2_O_5_, which exhibit improved resilience to volume changes during Zn^2+^ intercalation. It is worth noting that the effective integration of dopants into V_2_O_5_ cathodes, *via* either substitutional or interstitial pathways, requires a nuanced optimization of several interrelated parameters, including ionic radius compatibility, dopant oxidation state, local coordination environments, and the thermodynamics of defect formation. When these factors are appropriately balanced, doping can significantly enhance the structural integrity and Zn^2+^ diffusion kinetics of the V_2_O_5_ lattice, while also modulating the electronic density of states. Collectively, these modifications contribute to improved electrochemical performance and long-term cycling stability of V_2_O_5_ cathodes in AZIBs.

### 5.5. Challenges and future perspectives of interfacial engineering

Several approaches have been explored to overcome the intrinsic limitations of V_2_O_5_ cathodes in AZIBs. Unfortunately, most of these methods fall short in simultaneously enhancing ion transport, suppressing vanadium dissolution, and improving long-term electrochemical stability. Because these fundamental issues remain unaddressed, the overall performance and practical deployment of V_2_O_5_ cathodes are severely restricted. To tackle these challenges in a comprehensive way, interfacial engineering has emerged as a pivotal strategy, as it can tailor the cathode-electrolyte interface to promote faster Zn^2+^ diffusion, inhibit side reactions, and stabilize the V_2_O_5_ structure during cycling.^250,251^ Despite the potential of interface engineering to boost cathode functionality, its application faces significant hurdles, especially when subjected to extensive cycling. These operational constraints can give rise to mechanical and structural degradation. Popular choices for incorporating into V_2_O_5_ structures include conductive polymers such as polyaniline and PEDOT:PSS. These polymers are introduced to increase interlayer spacing and speed up the movement of Zn^2+^ ions. However, these polymer materials commonly exhibit poor mechanical durability. They can easily swell, peel away, or separate from the core active material during the recurring processes of Zn^2+^ insertion and removal. This ultimately undermines the integrity of the interface and causes a reduction in the capacity of the cathode over time.^252^ Another notable hurdle in interfacial engineering pertains to the restricted spatial reach of dopants and surface modifications. The method frequently produces inconsistent integration, particularly when dealing with heterogeneous doping or surface alterations involving polyvalent ions like Mg^2+^, Al^3+^, or Ti^4+^. These dopants frequently concentrate in specific locales, triggering strain buildup or phase separation.^253^ This irregular allocation fosters electrochemical variances, encouraging preferential Zn^2+^ inclusion at select spots. This targeted intercalation pattern may engender structural stress and fatigue during cycling, consequently jeopardizing the reversibility and endurance of the cathode substance.^253^ Dai *et al.*^254^ suggest that utilizing interphases, for example, metal oxide coatings or hybrid films frequently found in interfacial engineering, can be beneficial. Such layers might curb cathode material dissolution and improve the movement of Zn^2+^ ions. Nevertheless, their study uncovers some important drawbacks as well. Specifically, these intermediary layers are frequently lacking in the required chemical and mechanical robustness, a situation especially apparent within zinc sulphate (ZnSO_4_)-based electrolytes, known as for their mild acidity. In addition, using rigid coatings can present issues. They can impede ion transport, or, alternatively, face chemical deterioration as the battery cycles. These occurrences can generate passivation layers, consequently, raise the interfacial resistance and leading to a decline in the performance of the battery. Despite the increasing prevalence of sophisticated *in situ* and *operando* characterization tools, including X-ray absorption spectroscopy (XAS), transmission electron microscopy (TEM), and X-ray diffraction (XRD), achieving a thorough mechanistic grasp of interfacial dynamics within zinc-ion batteries has proven challenging. Key aspects, such as the impact of structural water, the structures of Zn^2+^ solvation, and the transient interfacial reactions during electrochemical operation, are not yet fully elucidated.^255^ This lack of understanding constraints the ability to rationally engineer improved interfacial architectures. To overcome these challenges, research is actively investigating hybrid interfacial designs that simultaneously prioritize mechanical resilience and efficient ion conduction.^255,256^ A particularly promising approach involves combining two-dimensional materials like MXenes and graphene oxide with ion-conducting polymers; this strategy aims to improve both the flexibility and structural durability of. Furthermore, atomic-level surface engineering methods like atomic layer deposition (ALD) allow for precise control over the thickness and composition of coatings, facilitating the creation of interfaces that are conformal and without defects. Supporting these experimental undertakings, computational techniques, specifically density functional theory (DFT) and machine learning, are becoming increasingly important for the prediction and optimization of interfacial materials. These tools offer valuable perspectives on factors such as dopant distribution, interfacial energies, and solvation characteristics, thereby accelerating the process of developing robust, high-performing interphases.^256^

## 6. Computational modeling

Computational modeling plays a critical role in the rational design and performance optimization of V_2_O_5_ cathodes for AZIBs. Among the various tools available, Density Functional Theory (DFT) is the most widely adopted due to its balance between accuracy and computational efficiency.^34^ DFT provides atomistic insights into key properties such as electronic structure, defect formation energies, redox potentials, and Zn^2+^ intercalation sites across different V_2_O_5_ polymorphs, including α-V_2_O_5_ and hydrated bilayer structures.^34,257^ Furthermore, DFT simulations have been instrumental in identifying energetically favourable Zn^2+^ intercalation pathways and predicting how dopants or defects influence the electronic conductivity and structural stability of the V_2_O_5_ lattice.^34,258^ Beyond static DFT calculations, *ab initio* molecular dynamics (AIMD) simulations serve as a powerful complementary tool to explore the real-time behaviour of V_2_O_5_ cathode materials under finite temperature and pressure conditions. AIMD offers critical insights into dynamic processes such as Zn^2+^ diffusion, lattice hydration, and phase transitions, which are essential for understanding the long-term stability and electrochemical behaviour of hydrated V_2_O_5_ in AZIBs. For example, Li *et al.* demonstrated through AIMD simulations how intercalated water molecules influence both the ion mobility and structural flexibility of bilayer V_2_O_5_ phases, thereby impacting Zn^2+^ ion transport and mechanical stability.^35,259^ However, despite its strengths, AIMD is computationally expensive and typically restricted to picosecond-scale trajectories and small supercells, limiting its applicability for studying long-term degradation or large-scale structural evolution. Therefore, AIMD is best suited for capturing fast, local events and is often used in conjunction with other methods, such as coarse-grained molecular dynamics or kinetic Monte Carlo simulations, to extend predictive insights across broader time and length scales.^260^ Machine learning (ML) approaches are increasingly being incorporated into V_2_O_5_ cathodes research as means to overcome limitations associated with traditional computational modeling, particularly those related to time and resource constraints of first-principles methods. ML models, especially neutral networks and graph-based algorithms, are trained on datasets generated from DFT or experimental results. Once trained, these models can quickly predict key properties such as phase stability, electronic structure, ionic conductivity, and the effects of dopants across broad compositional spaces and polymorphic phases.^261^ For examples, Zhang *et al.* employed a supervised learning approach to screen dopant combinations and nanostructured morphologies aimed at enhancing Zn^2+^ diffusion while mitigating structural degradation and vanadium active materials dissolution in aqueous media.^261,262^ Despite these advances, the field still faces critical challenges, particularly in ensuring high-quality training data, improving model interpretability, and enhancing the generalization of ML models to unseen material systems.^261^ Addressing these limitations is essential for achieving robust, data-driven design of next-generation V_2_O_5_-based cathodes. Another important approach for studying ion transport, stress development, and morphological changes at the electrode scale is multiscale modeling, which encompasses techniques such as phase-field modeling and continuum-level simulations.^34,256^ These models are valuable because they bridge the gap between atomistic-level insights (*e.g.*, from DFT or AIMD) and macroscale battery performance, offering a comprehensive understanding of electrochemical–mechanical coupling, phase transformations, and electrode degradation dynamics. One of their key advantages lies in their ability to simulate practical operation conditions over extended time and spatial scales.^34,262^ However, these models often rely on idealized assumptions and simplified material parameters, which can limit their predictive accuracy.^263^ An untapped opportunity lies in integrating experimental feedback, especially from *operando* techniques such as synchrotron XRD or XAS, to refine and validate model parameters, thereby enhancing their relevance to real-world systems.^264^

While computational modeling techniques such as DFT, AIMD, and multiscale simulations provide valuable insights into the structural, electronic, and transport properties of vanadium-based cathodes, several critical gaps remain. Notably, most models neglect electrolyte-related effects, including solvation shell dynamics, ion pairing, and electrolyte–cathode interfacial reactions, which are crucial for accurately capturing ion insertion and transport behaviour under realistic conditions.^265–267^ Additionally, these models typically do not account for long-term degradation mechanisms, such as structural collapse, water co-insertion, and irreversible phase transitions, which play significant roles in the performance fading of V_2_O_5_ cathodes over extended cycling. These degradation processes are underexplored computationally, partly due to the challenges in simulating phenomena that evolve over long timescales and involve complex, multicomponent environments.^266,268^ Future advances will likely depend on coupling atomistic models with *operando* experimental data and developing hybrid frameworks that integrate electrolyte effects and time-dependent degradation pathways.^267^

## 7. Conclusion and outlook

Vanadium pentoxide (V_2_O_5_) remains a leading candidate among materials for rechargeable AZIBs, owing to its rich redox chemistry, high theoretical capacity, and layered framework that accommodates Zn^2+^ and H_2_O co-intercalation. Progress over the past decade has demonstrated that rational modifications, ranging from ion pre-intercalation and conductive polymer hybridization to advanced nanostructuring and electrolyte engineering, can significantly enhance the stability, rate performance, and reversibility of V_2_O_5_-based systems. Despite these achievements, fundamental and application-specific challenges continue to impede the practical realization of V_2_O_5_ cathodes in commercial AZIB technologies. Foremost among these challenges is the structural instability that emerges from repeated Zn^2+^ insertion and extraction. This issue is not only linked to the intrinsic lattice fragility of V_2_O_5_ but also to local strain accumulation, irreversible phase transitions, and dissolution of vanadium species under prolonged cycling. These degradation processes are especially pronounced under deep discharge conditions, as required in stationary energy storage or load-levelling applications. Furthermore, significant voltage hysteresis persists due to multiphase behaviour, sluggish kinetics, and non-uniform ion distribution within the host framework. Addressing these phenomena requires a more nuanced understanding of the crystallographic evolution of V_2_O_5_ during operation, particularly under varying pH environments and electrolyte chemistries that modulate interfacial reactions and phase boundaries. Recent advances have begun to address these issues by introducing ultrathin protective layers, such as ion-conductive ceramics or 2D materials, which stabilize the V_2_O_5_-electrolyte interface without impeding ionic conductivity. Moreover, controlled doping and defect engineering, enabled by computational modeling, have proven effective in tuning local electronic environments and improving phase stability. In parallel, the development of high-concentration, pH-buffered, or “water-in-salt” electrolytes has shown promise in suppressing parasitic side reactions and expanding the electrochemical stability window. However, these strategies must be reconciled with cost, scalability, and computability with the zinc anode, particularly in full-cell configurations. The complex interplay between cathode structure, electrolyte composition, and anode interface evolution remains poorly understood and deserves greater attention, especially in view of electrolyte-driven cross-talk and dendrite-induced instability that can undermine long-term performance. To push the field forward, it is essential to integrate multiscale *in situ* and *operando* characterization techniques, including synchrotron XRD, *in situ* TEM, and Raman spectroscopy, with predictive modeling frameworks such as density functional theory (DFT), *ab initio* molecular dynamics (AIMD), and emerging machine learning (ML) algorithms. These tools not only allow direct observation of phase transitions, intercalation dynamics, and dissolution events, but also offer predictive insight into dopant behaviour, solvation environments, and reaction energetics across diverse compositions. Notably, ML-assisted screening methods trained on DFT datasets are increasingly capable of mapping vast chemical spaces, identifying promising dopant species, and forecasting stability trends in V_2_O_5_-derived materials.

Looking ahead, the rational design of hybrid cathodes, combining V_2_O_5_ with electronically conductive, mechanically robust, and chemically inert matrices such as MXenes, carbon nanotubes, or redox-active polymers such as quinone-based polymers, presents a promising avenue to bridge current limitations. However, material-level innovations must be evaluated in the context of broader system metrics, including energy density, cycle life, scalability of synthesis, and environmental footprint. Life-cycle assessment (LCA) and techno-economic modeling will be indispensable in translating laboratory breakthroughs into commercially relevant technologies. Ultimately, the transition of V_2_O_5_ cathodes from experimental systems to viable components in real-world AZIB applications will depend on a multidisciplinary approach that unifies fundamental electrochemistry, advanced materials science, scalable engineering, and system-level optimization. With continued innovation guided by deep mechanistic understanding and practical constraints, V_2_O_5_-based cathodes hold significant promise for powering the next generation of safe, sustainable, and high-performance applications energy storage systems.

## Conflicts of interest

There are no conflicts to declare.

## Data Availability

This review work does not involve the collection or analysis of new data.
